# Engineering *β*-ketoamine covalent organic frameworks for photocatalytic overall water splitting

**DOI:** 10.1038/s41467-023-36338-x

**Published:** 2023-02-03

**Authors:** Yan Yang, Xiaoyu Chu, Hong-Yu Zhang, Rui Zhang, Yu-Han Liu, Feng-Ming Zhang, Meng Lu, Zhao-Di Yang, Ya-Qian Lan

**Affiliations:** 1 Heilongjiang Provincial Key Laboratory of CO2 Resource Utilization and Energy Catalytic Materials, School of Material Science and Chemical Engineering, Harbin University of Science and Technology, Harbin, Heilongjiang 150080 People’s Republic of China; 2 School of Chemistry, South China Normal University, Guangzhou, Guangdong 510006 People’s Republic of China

**Keywords:** Coordination chemistry, Photocatalysis

## Abstract

Covalent organic frameworks (COFs) are an emerging type of crystalline and porous photocatalysts for hydrogen evolution, however, the overall water splitting activity of COFs is rarely known. In this work, we firstly realized overall water splitting activity of *β*-ketoamine COFs by systematically engineering N-sites, architecture, and morphology. By in situ incorporating sub-nanometer platinum (Pt) nanoparticles co-catalyst into the pores of COFs nanosheets, both Pt@TpBpy-NS and Pt@TpBpy-2-NS show visible-light-driven overall water splitting activity, with the optimal H_2_ and O_2_ evolution activities of 9.9 and 4.8 μmol in 5 h for Pt@TpBpy-NS, respectively, and a maximum solar-to-hydrogen efficiency of 0.23%. The crucial factors affecting the activity including N-sites position, nano morphology, and co-catalyst distribution were systematically explored. Further mechanism investigation reveals the tiny diversity of N sites in COFs that induces great differences in electron transfer as well as reaction potential barriers.

## Abstract (web summary)

Covalent organic frameworks (COFs) are an emerging type of crystalline and porous photocatalysts for hydrogen evolution. Here, the authors report a β-ketoamine COF by systematically engineering N-sites, architecture, and morphology for improved water splitting activity.

## Introduction

Overall water splitting into H_2_ and O_2_ driven by visible light is a friendly route to convert solar energy into renewable and green hydrogen energy^1,2^. Until now, numerous efforts have been devoted to develop inorganic semiconductor-based photocatalysts such as SrTiO_3_:Al^3^, Ta_3_N_5_^4^, LaMg_*x*_Ta_1−*x*_O_1+3*x*_N_2−__3*x*_^5^, and (Ga_1-*x*_Zn_*x*_)(N_1−__*x*_O_*x*_)^6^, while the types of organic photocatalysts for overall water splitting are still limited^7^. In recent years, covalent organic frameworks (COFs), as a new class of crystalline and porous organic materials assembled by the covalent connection of building blocks^8^, have shown excellent visible-light-driven photocatalytic activity for hydrogen evolution reaction (HER) in the presence of sacrificial electron donor due to its remarkable merits including: (1) Excellent visible light absorption ability and structural stability;^9^ (2) Porous structures with ordered pores favoring mass transfer and exposing active sites;^10^ (3) Structural diversity providing great opportunity to tune the band structures;^11^ (4) Regulable electron separation ability by designing donor-receptor molecule module at molecular level^12^. However, except for some covalent triazine frameworks (CTFs), the overall water splitting activity of COFs has not been revealed, and most of them only drive hydrogen and/or oxygen evolution half-reactions separately.

In fact, both graphitic carbon nitride (g-C_3_N_4_), CTFs, and polytriazine imides (PTIs), known as organic overall water splitting photocatalysts, share a similar structural feature of N-containing aromatic heterocycle^13–15^. Some pioneer works have illustrated that N atoms of aromatic heterocycle in COFs usually form substantial effect on HER activity by electronic and steric variations, which offers a direction for designing efficient active centers in COFs for enhancing HER activity^16^. Nevertheless, as we all know, oxygen evolution reaction (OER) with four electrons migration is rate-limiting step in overall water splitting, and rationally building robust OER active sites is critical in designing overall water splitting photocatalysts^17^. To unveil and recognize the OER sites in COFs, our group and other researchers have performed theoretical calculations on C_4_N-COFs and N_3_-COFs, which indicates that the sp^2^-hybridized C atoms in N-containing heterocycles or benzene rings neighboring the triazine section tend to be OER active sites^18,19^. These support N atoms in the heterocycle may not only benefit HER of organic photocatalysts, but also have the potential effect on OER active centers. Thus, rational constructing N-containing heterocycle in COFs and modulating the cooperative effect of N and C sites should be an effective strategy to realize overall water splitting activity for COFs photocatalysts.

**Fig. 1:**
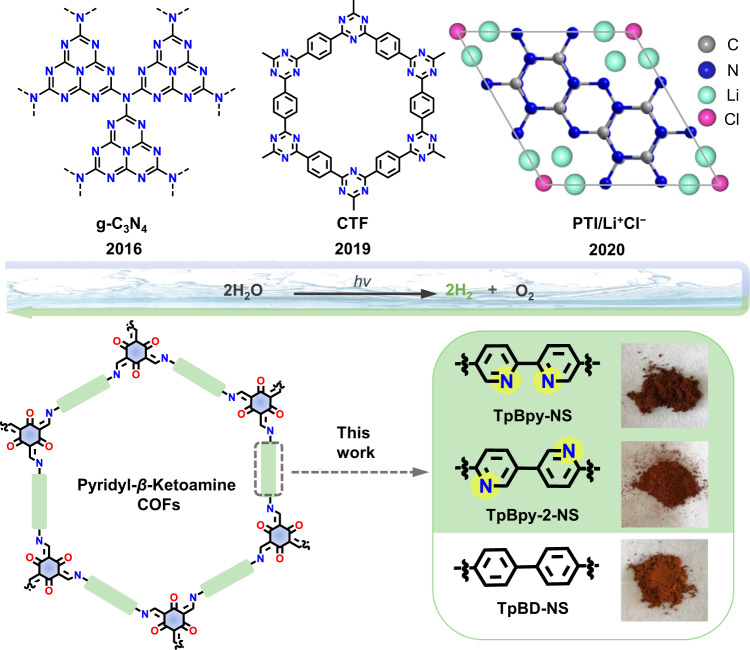
The brief development of organic photocatalysts for overall water splitting and related photocatalysts in this work. g-C_3_N_4_ graphitic carbon nitride, CTFs Covalent Triazine Frameworks, PTI Polytriazine Imide, COFs Covalent Organic Frameworks, respectively.

The *β*-ketoamine COFs, formed by reversible Schiff base reaction and irreversible enol-to-keto tautomerization, are a huge and representative category of highly stable COFs. In this work, we designed a series of *β*-ketoamine COFs with the same topology and similar atomic composition as a model system to investigate the effect of atoms at diverse positions on overall water splitting activity (Fig. 1). Three *β*-ketoamine COFs with and without bipyridine (Bpy) N sites were synthesized and in-situ incorporated ultra-small Pt nanoparticles (NPs) co-catalyst into the pores of COFs nanosheets (COFs-NS). As a result, both COFs containing Bpy segment, Pt@TpBpy-NS and Pt@TpBpy-2-NS, show the overall water splitting H_2_ and O_2_ production activity, while phenyl-structured Pt@TpBD-NS just drive H_2_ evolution half-reaction. The optimal visible-light-driven H_2_ and O_2_ amounts of Pt@TpBpy-NS are 9.9 and 4.8 μmol in 5 h, respectively, while 3.1 and 1.4 μmol for Pt@TpBpy-2-NS. The results of control experiments reveal the crucial factors that affect the overall water splitting activity including N-sites position, nano morphology, and Pt NPs distribution. The tiny difference of N sites in Bpy section of COFs and the active sites of overall water splitting were further investigated by theoretical calculation.

## Results

### Synthesis and characterizations of *β*-Ketoamine COFs nanosheets

**Fig. 2:**
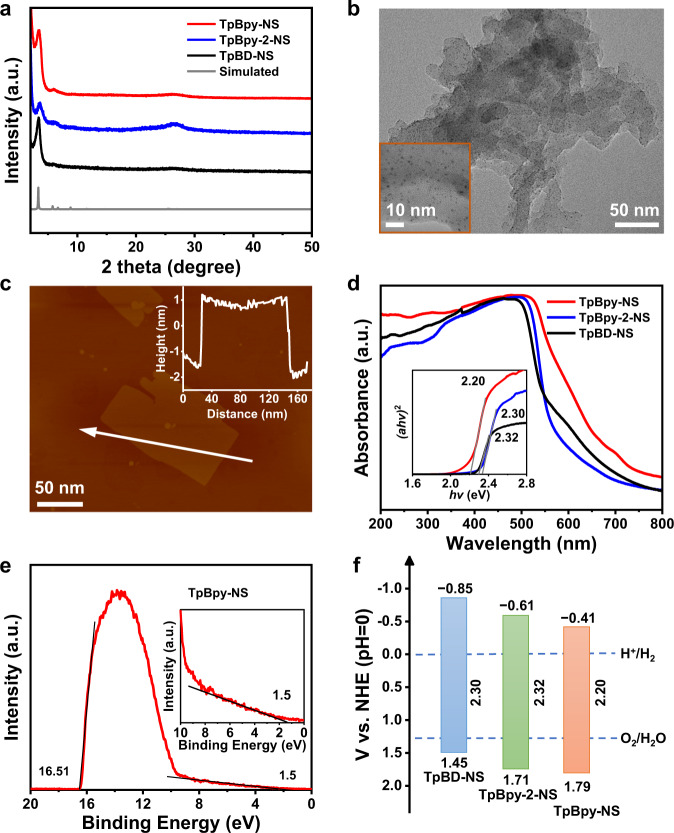
Characterizations of COFs-NS. **a** PXRD patterns of TpBpy-NS, TpBpy-2-NS, and TpBD-NS. **b** TEM image, **c** AFM image of Pt@TpBpy-NS. Scale bar: 50 nm. **d** UV–Vis DRS, and (*ahv*)^2^ versus *hv* curve of TpBpy-NS, TpBpy-2-NS, and TpBD-NS. **e** UPS spectra of TpBpy-NS. **f** Band structure of TpBpy-NS, TpBpy-2-NS, and TpBD-NS.

The TpBD-COF, TpBpy-2-COF, and TpBpy-COF, were synthesized by the reported methods with modification and exfoliated to COFs-NS by “top-down” route. With the aim of encapsulating highly dispersed Pt co-catalyst into the pores of COFs and COFs-NS, the as-synthesized ultra-small Pt NPs with the size around 1.1 nm were introduced to prepare Pt@COFs-NS and Pt@COFs photocatalysts by in-situ synthesis. The highly crystalline of COFs, Pt@COFs, COFs-NS and Pt@COFs-NS was confirmed via X-ray diffraction (PXRD) (Fig. 2a and Supplementary Figs. 1–3). All experimental PXRD patterns of these COFs perfectly match the diffraction of simulated AA stacking mode with high crystallinity^20^. The open channels in these COFs provide the opportunity of accommodating Pt NPs to prepare Pt@COFs-NS. In their Fourier infrared transform spectroscopy (FT-IR) spectra (Supplementary Figs. 4–6), the stretching vibration peak of C=O, C=C, and C–N could be observed that confirmed the formation of *β*-ketoamine structure by enol interconversion^21^. The permanent porosity of these COFs-NS was proved through N_2_ adsorption-desorption measurements (Supplementary Figs. 7–9). The corresponding Brunauer-Emmett-Teller (BET) surface areas are up to 1350, 1087, and 970 m^2^ g^−1^ for TpBpy-NS, TpBpy-2-NS and TpBD-NS, respectively^22^. The pore size distribution results also confirmed that the real pore sizes of these COFs-NS were centered on 2.1 nm (Supplementary Figs. 10–12). The BET surface areas of a series of Pt@COFs-NS are apparently lower than pristine COFs-NS and Pt/COFs-NS, supporting that Pt NPs were primarily encapsulated into the pores of COFs-NS^23^. The ZETA potentials of these COFs-NS are opposite to Pt NPs, meaning that Pt NPs could be tightly combined by COFs-NS matrix through electrostatic interaction (Supplementary Fig. 13)^24^. The X-ray photoelectron spectroscopy (XPS) for Pt@TpBpy-NS confirmed the co-existence of C, N, O, and metallic Pt (Supplementary Figs. 14–16). Moreover, the XPS N *1s* spectrum also supported that the Pt NPs were encapsulated in the pores of COFs-NS by the apparent change of binding energy after Pt loading^25^. Inductively coupled plasma emission spectroscopy (ICP-OES) confirmed that these Pt@COFs-NS with the adding amount of 5 wt% Pt NPs in in-situ synthesis have a similar Pt content, in the range of 1.19–1.28 wt% (Supplementary Table 1).

The transmission electron microscope (TEM) and energy dispersive X-ray (EDX) mapping images of Pt@COFs-NS confirmed that Pt NPs with an average diameter of 1.1 nm were uniformly distributed (Fig. 2b and Supplementary Figs. 17–20). It was observed that the Pt NPs distribution is obviously different between Pt@COFs-NS and Pt/COFs-NS. For Pt/COFs-NS obtained by the post-loading method, Pt NPs were enriched on the surface edge of COFs-NS with a bigger particle size (Supplementary Fig. 21)^26^. Moreover, atomic force microscope (AFM) measurement proves the nanosheet morphology of Pt@COFs-NS with a thickness in the range of 3–4 nm (Fig. 2c and Supplementary Figs. 22–23), corresponding to 8–12 layers stacking of COFs. The smooth surface of nanosheets supports that the Pt NPs were primarily distributed in the pores of COFs-NS. A typical Tyndall effect was observed for the solutions containing Pt@COFs-NS (Supplementary Fig. 24), confirming their colloidal structure^27^.

UV–Vis diffuse reflectance spectroscopy (DRS) shows that a series of Pt@COFs-NS exhibit similar light absorption compared to the pristine COFs-NS (Fig. 2d and Supplementary Figs. 25–27). The *E*_g_ of TpBpy-NS, TpBpy-2-NS and TpBD-NS were determined to be 2.20, 2.32, and 2.30 eV (inset in Fig. 2d), respectively^28^. The Mott-Schottky (M-S) curves of these COFs-NS were all positive slope, typical mode of n-type semiconductors (Supplementary Figs. 28–30). Accordingly, the conduction band (CB) positions of TpBpy-NS, TpBpy-2-NS and TpBD-NS were determined to be −0.41, −0.61, and −0.85 V (vs. NHE, pH = 0) with the corresponding valance band (VB) positions being 1.79, 1.71 and 1.45 V, respectively^29^. For Pt@COFs-NS, the CB are almost equal to their parent COFs-NS (Supplementary Figs. 31–33). Ultraviolet photoelectron spectroscopy (UPS) was further performed to more precisely detect their band positions (Fig. 2e and Supplementary Figs. 34–35). In this way, the VB energies of TpBpy-NS, TpBpy-2-NS, and TpBD-NS were calculated to be 6.2, 6.0, and 5.8 eV, respectively, corresponding to 1.80, 1.71, 1.40 V (vs. NHE, pH = 0)^30^, similar to the values obtained by M-S. Based on the above results, these COFs-NS are thermodynamically feasible for overall water splitting (Fig. 2f).

### Photocatalytic overall water splitting performances of COFs-NS

**Fig. 3:**
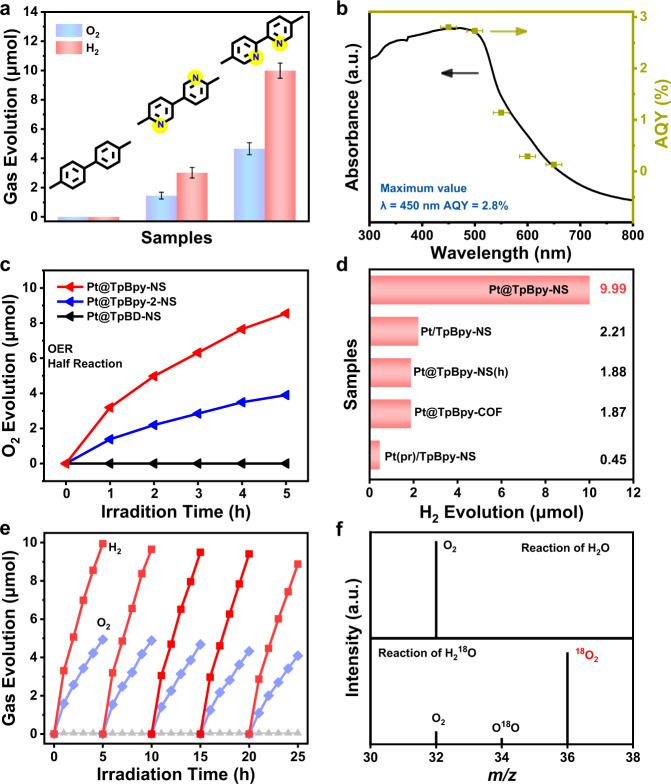
Photocatalytic performances of COFs-NS. **a** Overall water splitting activities comparison over Pt@COFs-NS. The error bar represents the standard deviation of the measurements. **b** Wavelength dependent AQY of overall water splitting for Pt@TpBpy-NS. The error bars indicate the incident wavelength with a full width at half maximum of 15 nm. **c** O_2_ evolution half-reaction rate over Pt@COFs-NS. **d** Overall water splitting activities comparison over TpBpy-COFs based catalysts. **e** Five-times cycle overall water splitting test of Pt@TpBpy-NS and **f** Mass spectra of Pt@TpBpy-NS in the photocatalytic reaction of H_2_O and H_2_^18^O.

The correlation between the loading amount of Pt NPs and overall water splitting performance was evaluated firstly. As shown in Fig. 3a and Supplementary Figs. 36–39, a volcanic linear relationship between photocatalytic activity and Pt content was observed for both Pt@TpBpy-NS and Pt@TpBpy-2-NS, and the adding amount of 5 wt% Pt NPs (real content of 1.2–1.3 wt% based on ICP-OES results) is optimal. However, no H_2_ or O_2_ can be detected for Pt@TpBD-NS with different Pt contents under visible or UV-Visible light irradiation. The optimized visible-light-driven H_2_ and O_2_ evolution amount in 5 h for Pt@TpBpy-NS are 9.9 and 4.8 μmol, respectively, while 3.1 and 1.4 μmol for Pt@TpBpy-2-NS. Notably, the overall water splitting activity of Pt@TpBpy-NS is superior to most of visible-light-driven organic photocatalysts and heterostructured systems reported previously (Supplementary Table 2). As presented in Supplementary Fig. 40, the H_2_ and O_2_ productions linearly increase with reaction time prolonging, and the ratio of H_2_ and O_2_ evolution is quite close to 2:1 stoichiometry of H_2_O splitting. The wavelength-dependent apparent quantum yield (AQY) of Pt@TpBpy-NS matches well with the DRS curves (Fig. 3b), indicating that the reaction was caused by the absorption of incident photons^31^. The highest AQY of 2.8% was observed at 450 nm for Pt@TpBpy-NS. The solar-to-hydrogen (STH) energy conversion efficiency of Pt@TpBpy-NS, evaluated under AM 1.5 G simulated sunlight (100 mW cm^−2^, 1 cm^2^) irradiated for 2 h, was determined to be 0.23%.

The comparisons of photocatalytic overall water splitting activity and the structural diversity of these three COFs-NS are shown in Fig. 3a. Two key factors impacting the overall water splitting activity in this system can be concluded. Firstly, bipyridine structure is essential for overall water splitting, because both Pt@TpBpy-NS and Pt@TpBpy-2-NS with bipyridine section exhibited excellent H_2_ and O_2_ evolution performance while Pt@TpBD-NS with biphenyl units did not show overall water splitting activity; Secondly, the overall water splitting activity also closely related to the position of N sites in the heterocyclic ring which can be deduced from the apparent difference of photocatalytic performances between Pt@TpBpy-NS and Pt@TpBpy-2-NS. Additionally, a broader visible light absorption range of Pt@TpBpy-NS than the other two COFs-NS should benefit its photocatalytic activity. However, the effect from the light absorption should be limited in comparison with the distinctness in structure and N-sites position, because Pt@TpBD-NS and Pt@TpBpy-2-NS exhibit similar light absorption but Pt@TpBD-NS have no overall water splitting activity.

The results of visible-light-driven hydrogen evolution half reaction confirmed that the H_2_ evolution amount of Pt@TpBpy-NS reached 1.7 mmol after irradiated for 5 h, which is 2.38 and 5.05 times higher than that of Pt@TpBpy-2-NS and Pt@TpBD-NS, respectively (Supplementary Figs. 41–42). The OER half reaction demonstrates that the Pt@TpBpy-NS and Pt@TpBpy-2-NS exhibited the O_2_ evolution amount of 8.55 and 4.05 μmol in 5 h, respectively (Fig. 3c). It is worthy of noting that no detectable O_2_ was produced in OER measurement for Pt@TpBD-NS. Thus, it is clear that the failed overall water splitting activity for Pt@TpBD-NS is ascribed to the hinder of OER process, probably due to its weak OER driving force and/or lacking appropriate OER active sites.

As shown in Fig. 3d, various control experiments imply that the unique architecture and morphology of Pt@COFs-NS in this work also play an important role in photocatalytic activity. We confirmed that bulk COFs without nanosheet morphology barely drive overall water splitting reaction with photoreduced Pt as cocatalyst (Pt(pr)/COFs). Then both Pt(pr)/TpBpy-NS and Pt/TpBpy-NS obtained by post synthesis with Pt NPs and TpBpy-NS could drive overall water splitting reaction with weak activities of 0.45 and 2.21 μmol H_2_ production in 5 h, respectively. Moreover, the activities of the Pt@TpBpy-COF with bulk COFs and Pt@TpBpy-NS(h) with agglomeration of COFs-NS were far less than that of Pt@TpBpy-NS. These comparisons prove that the nanosheet morphology of COFs and the architecture with ultra-small Pt NPs encapsulated in the pores of COFs are crucial for the high overall water splitting activity of the resultant materials. As reported, the nanosheets morphology of 2D COFs could benefit the charge separation and transfer^32^. And Pt NPs uniformly distributed in pores of COFs are more efficient for extracting photogenerated electrons to Pt NPs^33^.

Further, 600 min continuous overall water splitting experiment for Pt@TpBpy-NS confirmed that the production of H_2_ and O_2_ could keep increasing (Supplementary Fig. 43), although the increase rate has slightly decreased probably due to the back-reaction. As shown in Fig. 3e, only a small decay (<5%) was observed after five cycle reactions and the ratio of H_2_ and O_2_ still keeps 2:1. The results of PXRD, FT-IR spectra, XPS spectra, TEM images, and ICP-OES measurements for Pt@TpBpy-NS (Supplementary Figs. 44–48) after long-term reaction were similar to the fresh sample, indicating its stability of structure and morphology in photocatalytic reaction^34,35^. The water splitting performed using H_2_^18^O are shown in Fig. 3f, confirmed that O_2_ was generated by photocatalytic water splitting and not from other sources.

### Photophysical and photochemical properties study of COFs-NS

**Fig. 4:**
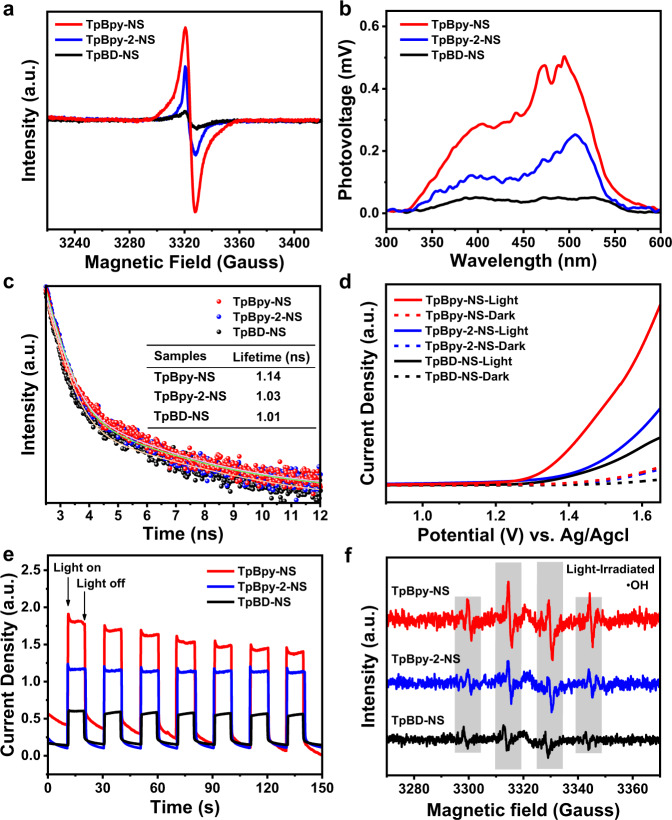
Photophysical and photochemical properties of COFs-NS. **a** EPR spectra under illumination, **b** SS-SPS spectra, **c** time-resolved PL spectra, **d** *I*−*V* curves in dark and under light irradiation, **e** transient photocurrent spectra, and **f** EPR spectra of •OH radical trapped by DMPO over TpBpy-NS, TpBpy-2-NS, and TpBD-NS.

The electron paramagnetic resonance (EPR) peaks of all COFs-NS under light are apparently higher than that in the dark field (Supplementary Figs. 49–51). In comparison, TpBpy-NS showed a stronger EPR signal than others (Fig. 4a), demonstrating its superiority in the production of active electrons. As shown in Fig. 4b, TpBpy-NS and TpBpy-2-NS have obvious surface photovoltage spectroscopy (SPS) responses, while the response from TpBD-NS was negligible. As shown in Fig. 4c and Fig. S52, the photoluminescence emission intensity of TpBpy-NS was largely quenched and possessed the longest average lifetime (Aτ), meaning that TpBpy-NS has the longest carrier diffusion time^36,37^. Furthermore, electrochemical impedance spectroscopy (EIS) implies that TpBpy-NS has the smallest charge transfer resistance in this system (Supplementary Fig. 53)^38^. Besides, the linear sweep voltammetry (LSV) measurements show that TpBpy-NS has reduced onset overpotentials for both H_2_ and O_2_ evolution (Fig. 4d and Supplementary Fig. 54), which is consistent with photocatalytic half-reaction results^39^. The best charge carrier separation ability of TpBpy-NS was also supported by transient photocurrent spectra (Fig. 4e)^40^. All the above analyses proved that the existence and rational position of pyridine N atoms in the system could promote charge separation and migration. As shown in Fig. 4f, four consecutive EPR characteristic peaks related to the •OH were observed for these COFs-NS with radical trapping agent. However, the peak intensity of TpBpy-NS was stronger than that of TpBpy-2-NS and TpBD-NS, illustrating TpBpy-NS is more feasible to drive oxidation reaction of •OH to O_2_^41^. Although TpBD-NS has a more negative reduction potential, its ^•^O_2_^−^ signals that represent the reduction performance were still not as high as TpBpy-NS (Supplementary Fig. 55), which is consistent with their HER activity.

### DFT theoretical calculation

**Fig. 5:**
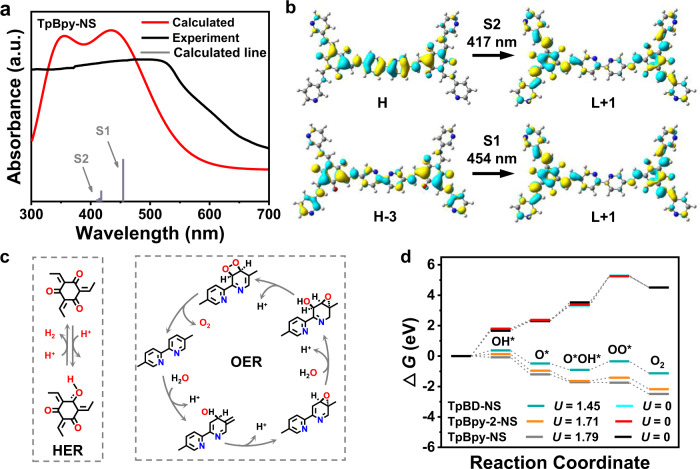
DFT calculations and proposed schematic mechanism of TpBpy-NS. **a** UV–Vis absorption spectra of TpBpy-NS compared with TD-DFT calculated fragment. **b** The TD-DFT calculated electronic transition of TpBpy-NS. **c** The possible process of HER on Tp segment and OER via dual-site process on Bpy segment in TpBpy-NS. **d** The comparison of calculated Gibbs free energy change for C2d paths of OER for TpBD-NS, TpBpy-2-NS and TpBpy-NS at pH = 7.

The systemic theoretical calculations based on density functional theory (DFT) were carried out on a series of *β*-ketoamine COFs (see calculation details in Supplementary Information and Supplementary Data 1). Time-dependent density functional theory (TD-DFT) calculation was carried out for model structures of TpBpy-2-NS and TpBpy-NS in order to acquire the charge transfer characteristic. The calculated UV–Vis absorption spectra shown in Fig. 5a are in good agreement with the experimental results. For TpBpy-NS, the maximum absorption peak comes from the transition of S_0_ → S_1_ and S_0_ → S_2_. The excited state S_1_ could be described as a linear addition of main configurations HOMO-3→LUMO + 1 and HOMO → LUMO, and the excited state S_2_ is mainly contributed by main configurations HOMO → LUMO + 1 and HOMO-1→LUMO. Figure 5b and Supplementary Fig. 56 give the contour surfaces of the frontier orbitals HOMO-3, HOMO-1, HOMO, LUMO and LUMO + 1 relevant to the maximal absorption. Obviously, both transitions of HOMO-3→LUMO + 1 and HOMO → LUMO + 1 show the intramolecular charge transfer (ICT) characteristic, that is the electrons transfer from Bpy to TP segment. For TpBpy-2-NS, the calculated UV–Vis absorption spectra and the contour surfaces of the frontier orbitals relevant to the maximal absorption are given in Supplementary Fig. 57. By the similar analysis, it can be found that the same ICT characteristic for TpBpy-2-NS but the electrons transfer from Bpy to Tp segment is not evident. Based on the obvious ICT mode of TpBpy-NS, the photogenerated electrons transferred to the O sites on Tp segments would induce subsequent HER (Supplementary Fig. 58 gives the Δ*G*_H*_ comparison of O and C as HER sites), the holes accumulations on C sites on Bpy segments would induce the OER process.

The optimized unit cells for these COFs are shown in Supplementary Fig. 59 and the sp^2^-hybridized C active sites (C1, C2, and C3) for OER were marked. The possible processes of HER and two possible OER circles including single- and dual-site processes with the formation of OOH* and O*OH* were evaluated in Fig. 5c and Supplementary Fig. 60. The OER on C1 and C3 sites are single-site paths (named as C1s and C3s, respectively), and OER on C2 site includes both single-site and dual-site paths (C2s and C2d). Four OER paths C1s, C2s, C2d, and C3s for TpBpy-NS and TpBpy-2-NS at pH = 7 were presented in Supplementary Figs. 61 and 62. The results demonstrate that the rate-determining step for paths C1s, C2s, and C3s is OOH* formation while the step for path C2d is OO* formation at pH = 7 and *U* = 0. The activation energy of the rate-determining step for single-site process (Δ*G*_OOH*_–Δ*G*_O*_) is obviously higher than that for dual-site process (Δ*G*_OO*_–Δ*G*_O*OH*_). Thus, the C2d path is considered as the most reactive path in view of its energetic favorable than others. The comparison of the free energy changes for each step in C2d paths for these COFs was performed with or without the experimental light-induced bias potential *U* at pH = 7 (Fig. 5d). As a result, the Δ*G* of each step is in the sequence of TpBpy-NS < TpBpy-2-NS < TpBD-NS when *U* and pH = 7 are applied. All steps of C2d path for TpBpy-NS is downhill at *U* = 1.79 V and pH = 7. The calculated results of excited states and OER pathway further verified and explained the excellent photocatalytic over water splitting.

## Discussion

Rational incorporating N-heterocycle is critical for realizing water splitting in this system, as we observed that Pt@TpBpy-NS and Pt@TpBpy-2-NS, both containing Bpy segment, show water splitting activity, while phenyl-structured Pt@TpBD-NS just drive H_2_ evolution half-reaction. Meanwhile, the N-sites position in Bpy section of COFs still significantly affects its activity, which leads to the diverse water splitting performance between Pt@TpBpy-NS and Pt@TpBpy-2-NS. Besides, it was confirmed that the nano sheet morphology of COFs and the incorporated ultra-small Pt NPs in the pores of COFs are key factors for enhancing water splitting activity in experiment. More deep insight for the overall water splitting mechanism suggests that the electrons transfer from Bpy to Tp section is more efficient in TpBpy-NS than that in TpBpy-2-NS, and a C2d are the optimal OER path. The conclusion of this work should be effective for other types of COFs and the atomic level N-site controllable strategy demonstrated will provide cutting-edge research inspirations for the design of organic catalysts with excellent redox activity.

## Methods

### Material preparation

All chemicals and reagents were of analytical grade and used without further purification. Deionized water was used throughout the experiments.

### Synthesis of Pt NPs

0.5 mL H_2_PtCl_6_·6H_2_O ethylene glycol solution (10 mg mL^−1^) was measured in Schlenk tube, and 0.11 g polyvinyl pyrrolidone (PVP), 23.5 mL anhydrous ethylene glycol, 1.0 mL NaOH ethylene glycol solution (0.2 mol L^−1^) were added into above solution. The mixture solution was sonicated for 5 min, and then vacuumed to a pressure value of 5.0 mbar. The vacuum tube containing the mixed solution was heated with a microwave oven (800 W) until the solution turned brownish yellow and cool to room temperature to obtain PVP protected Pt NPs. Subsequently, an appropriate amount of the solution was precipitated with acetone, and then collected by centrifugation at 8000 rpm for 5 min. Then the sample was washed with acetone and hexane to remove excess free PVP and then redispersed in Dimethylacetamide (DMAc) or Dimethylformamide (DMF) to obtain a colloidal solution of Pt NPs with a concentration of about 2.2 mg mL^−1^. The average size of the synthesized Pt NP is about 1.3 nm.

### Synthesis of Pt(*X*%)@TpBpy-COF

The 1,3,5-triformylphloroglucinol (Tp) (42.0 mg, 0.2 mmol), 2,2’-bipyridine-5,5’-diamine (Bpy) (55.8 mg, 0.3 mmol), 1 mL of o-dichlorobenzene (o-DCB), 3 mL of mixed solution composed of DMAc and Pt-DMAc dispersion (Pt-DMAc ratio = 0, 11.6, 23.7, 35.7, 47.3, 59.3, 71, 83 v/v%), 0.4 mL of 6.0 M aqueous acetic acid (AcOH) was charged in a pyrex tube. This mixture was sonicated for 10–15 min in order to get a homogenous dispersion. The tube was frozen at 77 K (liquid N_2_ bath) and degassed by three freeze-pump-thaw cycles. Then the tube was sealed off and heated at 120 °C for 3 days. A dark red colored precipitate formed was collected by centrifugation or filtration and washed with 5–6 times with DMAc, water and then acetone. The powder collected was then solvent exchanged with acetone three times and then freeze-drying to give a dark red colored powder in 79% isolated yield.

### Synthesis of Pt(*X*%)@TpBpy-2-COF

The Tp (42.0 mg, 0.2 mmol), 3,3’-bipyridine-6,6’-diamine (Bpy-2) (55.8 mg, 0.3 mmol), 1 mL of o-DCB, 3 mL of mixed solution composed of DMAc and Pt-DMAc dispersion (Pt-DMAc ratio = 0, 11.6, 23.7, 35.7, 47.3, 59.3, 71, 83 v/v%), 0.4 mL of 1.0 M AcOH was charged in a pyrex tube. This mixture was sonicated for 10–15 min in order to get a homogenous dispersion. The tube was frozen at 77 K (liquid N_2_ bath) and degassed by three freeze-pump-thaw cycles. Then the tube was sealed off and heated at 120 °C for 3 days. A dark red colored precipitate formed was collected by centrifugation or filtration and washed with 5–6 times with DMAc, water and then acetone. The powder collected was then solvent exchanged with acetone three times and then freeze-drying to give a dark red colored powder in 80% isolated yield.

### Synthesis of Pt(*X*%)@TpBD-COF

The Tp (42.0 mg, 0.2 mmol), Benzidinea (BD) (55.2 mg, 0.3 mmol), 3 mL of mixed solution composed of DMF and Pt-DMF dispersion (Pt-DMF ratio = 0, 58.7 v/v%), 0.35 mL of 3.0 M AcOH was charged in a pyrex tube. This mixture was sonicated for 10–15 min in order to get a homogenous dispersion. The tube was frozen at 77 K (liquid N_2_ bath) and degassed by three freeze-pump-thaw cycles. Then the tube was sealed off and heated at 120 °C for 3 days. A light red colored precipitate formed was collected by centrifugation or filtration and washed with 5–6 times with DMF, water and then acetone. The powder collected was then solvent exchanged with acetone three times and then freeze-drying to give a light red colored powder in 80% isolated yield.

### Synthesis of COFs-NS or Pt@COFs-NS

The 50 mg COFs or Pt(*X*%)@COFs was take into 100 mL water and ultrasonic for 30 min to obtain the nanosheet solution, then centrifuged at 3000 rpm, the upper layer liquid was collected and freeze-dry to obtain COFs-NS or Pt(*X*%)@COFs-NS.

### Synthesis of Pt/COFs-NS

The 15 mg of COFs-NS was added into the aqueous solution (15 mL) which containing the Pt solution (0.34 mL). And the mixture was sonicated for 10 min and stirred under vacuum for 1 h. Finally, the mixture was washed with plenty of water and freeze-dry.

### Synthesis of Pt(pr)/COFs-NS

The 15 mg of COFs-NS was added into the aqueous solution (15 mL), then 0.15 mL H_2_PtCl_6_•6H_2_O (8 mg mL^−1^) aqueous solution was added into the mixture and the solution was keep irradiation under the Xe lamp for 30 min. Finally, the mixture was washed with plenty of water and freeze-dry.

### Synthesis of Pt@TpBpy-NS(h)

The 50 mg Pt(*X*%)@COFs was take into 100 mL water and ultrasonic for 30 min to obtain the nanosheet solution, then centrifuged at 3000 rpm, the upper layer liquid was collected and processed by vacuum drying at 150 °C to obtain Pt@TpBpy-NS(h).

### Characterizations

The powder X-ray diffraction (PXRD) spectra were recorded on a Bruker D8 X-ray diffractometer with Cu Kα radiation (λ = 1.5405 Å) at 45 kV, 200 mA. Fourier transform infrared (FT-IR) spectra were recorded in Spectrum 100 spectrometer. Fourier transform infrared (FT-IR) spectra were recorded in Spectrum 100 spectrometer. The ZETA potential was recorded using a Zeta Potential Analyzer (DLS) with the instrument model Zetasizernano, and water was used as a dispersant. X-ray photoelectron spectroscopy (XPS) were recorded using a Thermo Escalab 250 instrument, equipped with an Al Kα microfocused X-ray source and the C1s peak at 284.6 eV as internal standard. Using inductively coupled plasma optical smission spectrometer (ICP-OES) model PerkinElmer 8300 to determine the content of metal element. Using JEM-2100 electron microscope observe the nano-scale morphology and lattice fringes of samples. Using Bruker Dimension ICON atomic force microscope (AFM) observe the surface morphology, thickness and roughness test of samples. Optical properties were measured by UV–Vis-NIR diffuse reflectance spectroscopy (UV–Vis-NIR DRS) spectroscopy (Lambda 35 spectrometer). The UPS analyzes the work function and the valence band structure of the conductor, the instrument model was Thermo ESCALAB 250XI PHI5000 VersaProbe III, and added a −5 eV bias to the sample during the test. The H_2_^18^O tracing experiment was analyzed using gas chromatography-mass spectrometry (7890A and 5975C, Agilent Technologies). The Eelectron paramagnetic resonance (EPR) spectrum was used to directly track the unpaired electronic situation, measuring instrument model: Bruker EMXplus. In the process of testing hydroxyl radicals and superoxide radicals, 5, 5-dimethyl-1-pyrroline N-oxide (DMPO) was used as a trapping agent. The surface photovoltage spectroscopy (SPS) measurements were carried out with a home-built apparatus equipped with a lock-in amplifier (SR830) synchronized with alight chopper (SR540). Photoluminescence spectrum (PL) were recorded using SPEX Fluorolog-3 spectrofluorometer with an excitation wavelength of 370 nm. Time resolved florescence decay spectrum were recorded using FLS920 with excitation of 370 nm and emission of 600 nm.

### Photoelectrochemical studies

The Mott-Schottky plot, electrochemical impedance spectra (EIS), linear sweep voltammetry (LSV) and transient photocurrent spectra was recorded on the CHI660E electrochemical workstation with a standard three-electrode system with the photocatalyst-coated ITO as the working electrode, Pt plate as the counter electrode, and a saturated silver chloride electrode as a reference electrode. A 0.5 M Na_2_SO_4_ solution was used as the electrolyte. The as-synthesized samples (2 mg) were added into 1 mL ethanol and 10 μL Nafion mixed solution, and the working electrodes were prepared by dropping the suspension (200 μL) onto an ITO glass substrate electrode surface and dried at room temperature. A 300 W Xenon lamp with a 420 nm cut-off filter was used as the light source during the measurement.

### Photocatalytic overall water splitting measurements

The 15 mg catalyst and 50 mL deoxygenated H_2_O was loaded in a sealed system, which was mainly composed of a quartz tube and a sealed system. Before the photocatalytic test, the device was purged with Ar flow to remove air, and then the reaction system was evacuated for 30 min to remove dissolved gases and ensure vacuum conditions. The photocatalytic overall water splitting experiment was carried out at 10 °C, and a 300 W Xe lamp with a cut-off filter (>420 nm) was used as the light source to achieve visible light irradiation. A gas chromatograph (GC7920, thermal conductivity detector (TCD), Ar carrier gas) was used to determine the product.

### Photocatalytic H_2_ evolution measurements

The 15 mg catalyst and 50 mL deoxygenated H_2_O containing 100 mg of sodium ascorbate (SA) as sacrificial electron donor was loaded in a sealed system, which was mainly composed of a quartz tube and a sealed system. Before the photocatalytic test, the reaction system was evacuated for 30 min to remove dissolved gases and ensure vacuum conditions. The photocatalytic overall water splitting experiment was carried out at 10 °C, and a 300 W Xe lamp with a cut-off filter (>420 nm) was used as the light source to achieve visible light irradiation. A gas chromatograph (GC7920, thermal conductivity detector (TCD), Ar carrier gas) was used to determine the product.

### Photocatalytic O_2_ evolution measurements

The 15 mg catalyst and 50 mL deoxygenated H_2_O containing 0.01 M AgNO_3_ and 0.1 g La_2_O_3_ as electron acceptor and a pH buffer agent respectively was loaded in a sealed system, which was mainly composed of a quartz tube and a sealed system. Before the photocatalytic test, the reaction system was evacuated for 30 min to remove dissolved gases and ensure the vacuum conditions. The photocatalytic overall water splitting experiment was carried out at 10 °C, and a 300 W Xe lamp with a cut-off filter (>420 nm) was used as the light source to achieve visible light irradiation. A gas chromatograph (GC7920, thermal conductivity detector (TCD), Ar carrier gas) was used to determine the product.

### AQY determination

The apparent quantum Yield (AQY) for hydrogen evolution was measured under the illumination of a 300 W Xe lamp with different bandpass filters for 5 h. The reported AQY values here are maximum attainable results after varying the amounts of photocatalysts used, the light intensity, and the light absorption areas. The AQY values for water splitting involving one-step photo-excitation were calculated using the following Eq. (1):

1 $${{{{{\rm{AQY}}}}}}(\%)=\frac{2\times {{{{{\rm{the}}}}}}\,{{{{{\rm{number}}}}}}\,{{{{{\rm{of}}}}}}\,{{{{{\rm{evolved}}}}}}\,{{{{{{\rm{H}}}}}}}_{2}\,{{{{{\rm{molecules}}}}}}}{N}\times 100\%$$

For example, at λ0 = 450 nm, for the average intensity of irradiation was determined to be 2.86 mW cm^−2^ by a spectroradiometer and the irradiation area was controlled at 7 cm^2^. The number of incident photons (*N*) is 8.17 × 10^20^. Used 80 mg Pt@TpBpy-NS as the catalyst, the amount of H_2_ molecules generated in 5 h was 19 μmol. Thus, the AQY calculated from above equation is ~2.8 %.

### STH determination

The solar-to-hydrogen conversion efficiency (STH) via the following Eq. (2):

2 $${{{{{\mathrm{STH}}}}}}=\frac{{{R}}({{{{{\mathrm{H}}}}}}_{2})\times {\varDelta} {{{{{\mathrm{G}}}}}}_{r}}{P}\times {S}\times 100 {\%}$$

Where *R*(H_2_), Δ*G*_r_, *P*, and *S* represent the rate of hydrogen evolution, the Gibbs energy for the reaction (H_2_O (l) → H_2_ (g) + 1/2 O_2_ (g)), the energy intensity of the AM 1.5 G solar irradiation (100 mW cm^−2^) and the irradiated sample area (7 cm^2^), respectively. For example, when using 80 mg Pt@TpBpy-NS catalyst, the irradiated sample area is 7 cm^2^ during 2 h of illumination. During the photocatalytic reaction, 44 μmol H_2_ was generated. Thus, the STH calculated from above equation is ~0.23 %.

### Computational methods

The systemic theoretical calculations were carried out for four C, N-based 2D-COFs. All calculations are performed based on density functional theory (DFT) calculations as implemented in Vienna ab initio simulation package (VASP) with projected augmented wave (PAW) method^42^. The exchange correlation energy was described with the Perdew-Burke-Ernzerhof (PBE) functional of the generalized gradient approximation (GGA)^43^. The energy cutoff is set to be 500 eV. A vacuum region of about 14 Å is used to eliminate interaction of layers. The k-point sampling of the Brillouin zone was obtained using a 2 × 2× 1 grid for all C, N-based 2D-COFs. Both lattice constants along the periodic direction and atomic positions are fully relaxed until the convergence criteria of energy and force is less than 10^−5^ eV and 0.01 eV Å^−1^, respectively. The free energy change (Δ*G*) of HER and OER calculated based on the computational hydrogen electrode (CHE) model developed by Nørskov et al.^44^. TDDFT (time-dependent density functional theory) was employed to predict excited state energies and properties to obtain the information of charge transfer^45^.

## Supplementary information

Supplementary Information

Description of Additional Supplementary Files

Supplementary Data 1

## Data Availability

Source data are provided with this paper. The data used in this study are presented in the text, Supplementary Information and Source Data. Additional data and information are available from the corresponding author on request. Source data are provided with this paper.

## Source data

Source Data
