# Supplementary material for: Engineering β-ketoamine covalent organic frameworks for photocatalytic overall water splitting
Source: Nat Commun. 2023 Feb 3;14:593. doi: 10.1038/s41467-023-36338-x (PMC9898260; doi:10.1038/s41467-023-36338-x)
Supplement: Supplementary file 1 — Supplementary Information [file 41467_2023_36338_MOESM1_ESM.pdf]

Supplementary Information

Engineering *β*-Ketoamine Covalent Organic Frameworks for Photocatalytic Overall Water Splitting

Yan Yang[1,†], Xiaoyu Chu[1,†], Hong-Yu Zhang[1,†], Rui Zhang[1], Yu-Han Liu[1], Feng-Ming Zhang[1,*], Meng Lu[2], Zhao-Di Yang[1,*] & Ya-Qian Lan[2,*]

[1]Heilongjiang Provincial Key Laboratory of CO_2 Resource Utilization and Energy Catalytic Materials, School of Material Science and Chemical Engineering, Harbin University of Science and Technology, Harbin, Heilongjiang 150080 (P. R. China).
[2]School of Chemistry, South China Normal University, Guangzhou, Guangdong 510006 (P. R. China).
[†]These authors contributed equally: Yan Yang, Xiaoyu Chu, Hong-Yu Zhang.
*e-mail: zhangfm80@163.com; yangzhaodi@163.com; yqlan@m.scnu.edu.cn.

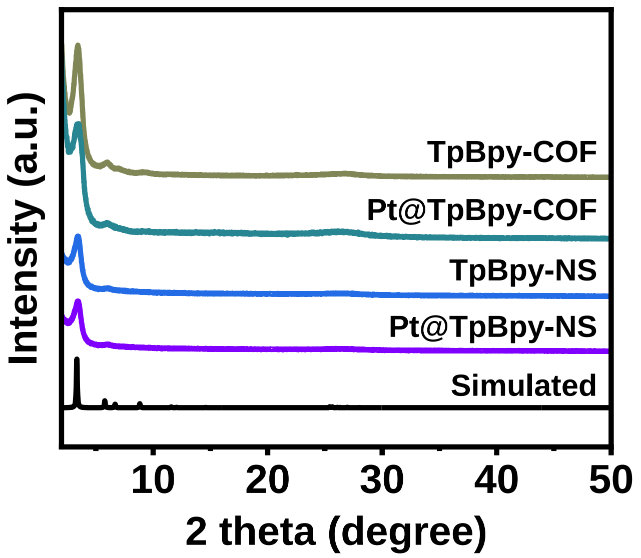

Supplementary Figure 1. PXRD patterns of TpBpy based materials.

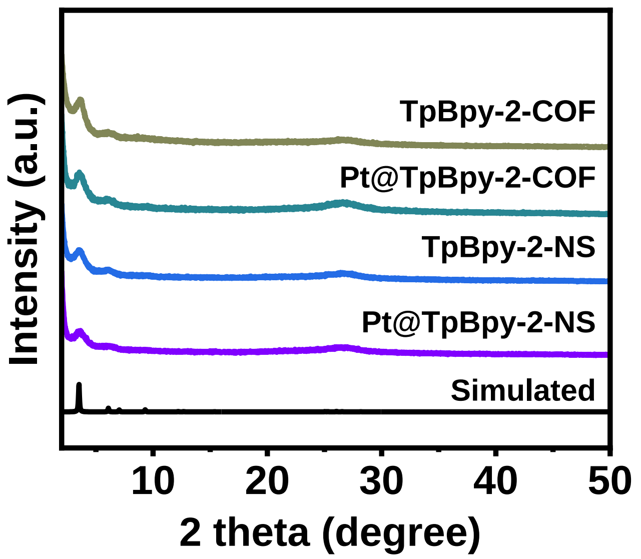

Supplementary Figure 2. PXRD patterns of TpBpy-2 based materials.

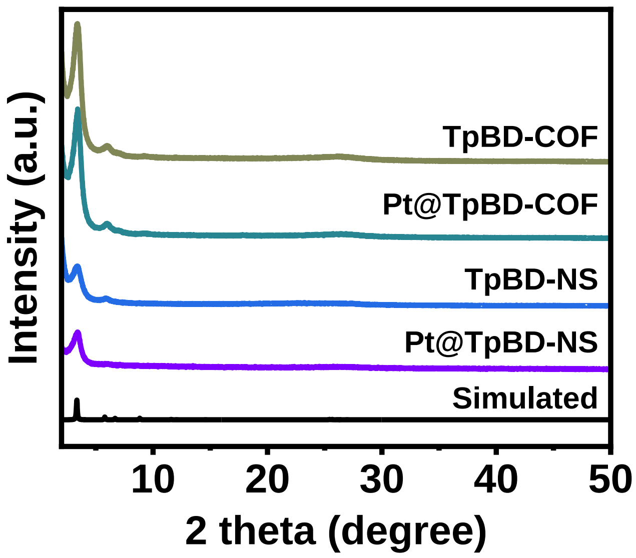

Supplementary Figure 3. PXRD patterns of TpBD based materials.

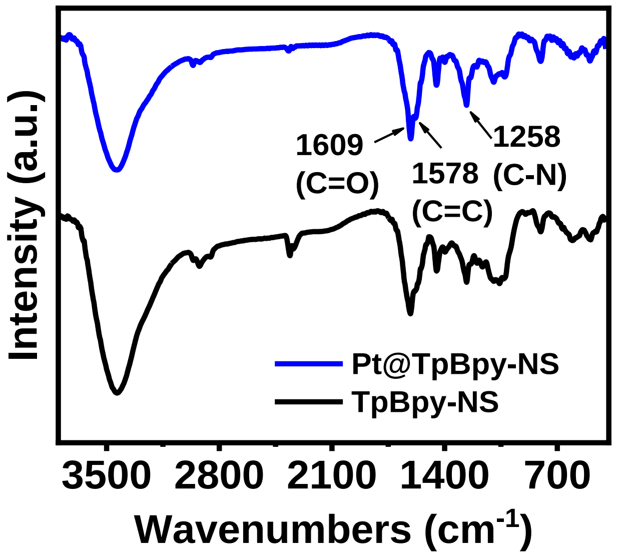

Supplementary Figure 4. Comparison of FT-IR spectra of TpBpy-NS and Pt(5%)@TpBpy-NS.

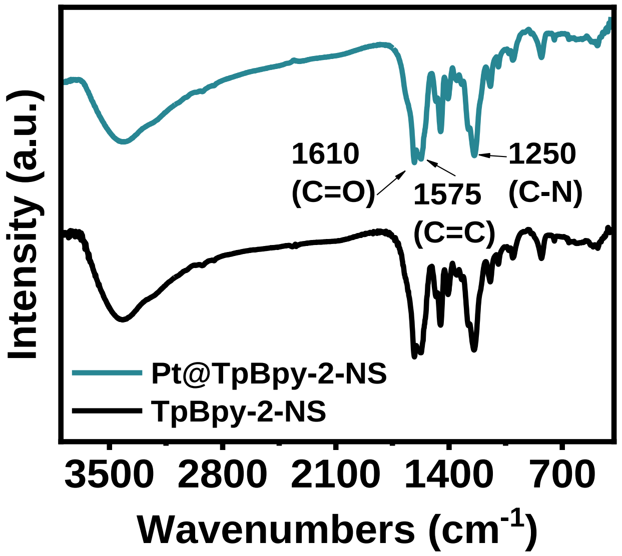

Supplementary Figure 5. Comparison of FT-IR spectra of TpBpy-2-NS and Pt(5%)@TpBpy-2-NS.

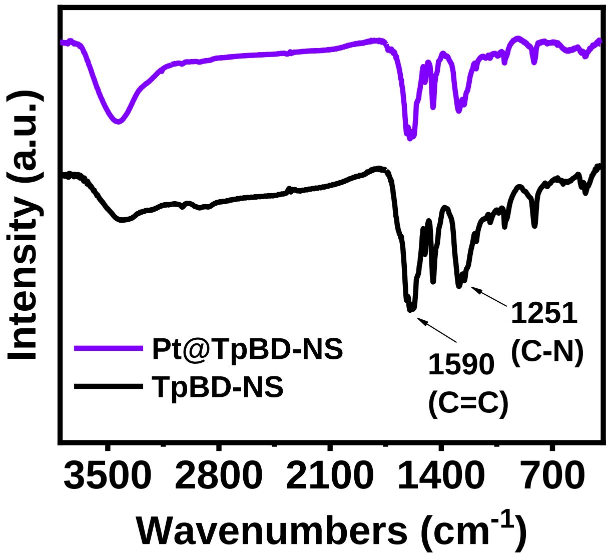

Supplementary Figure 6. Comparison of FT-IR spectra of TpBD-NS and Pt(5%)@TpBD-NS.

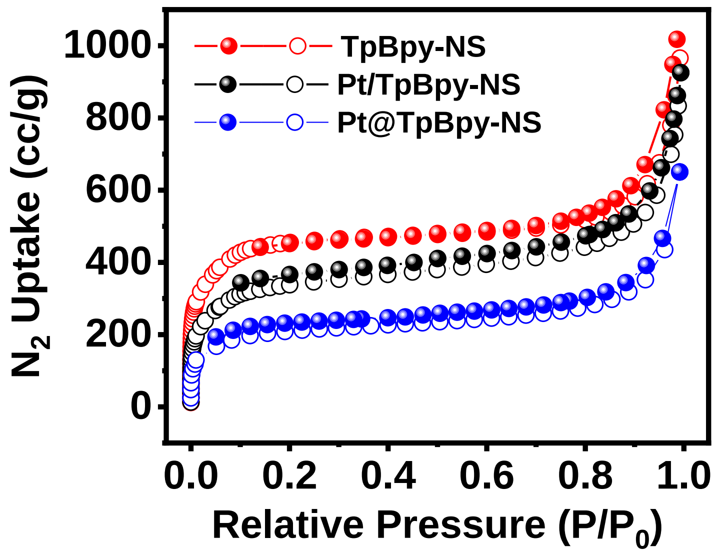

Supplementary Figure 7. Comparison of Nitrogen sorption curves of TpBpy-NS based materials measured at 77 K.

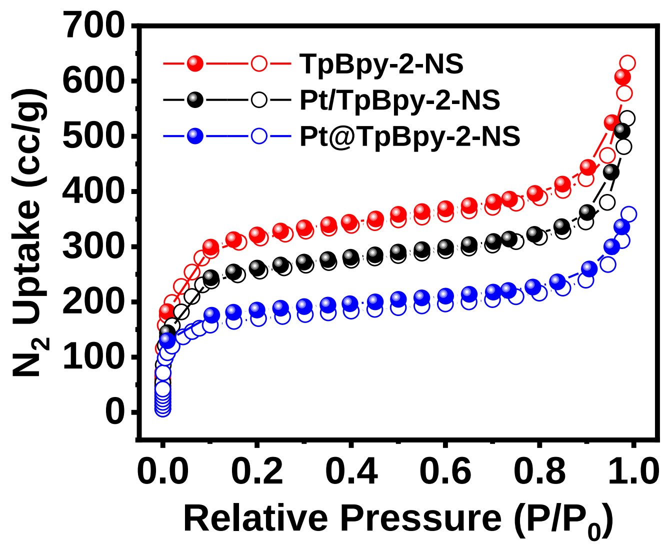

Supplementary Figure 8. Comparison of Nitrogen sorption curves of TpBpy-2-NS based materials measured at 77 K.

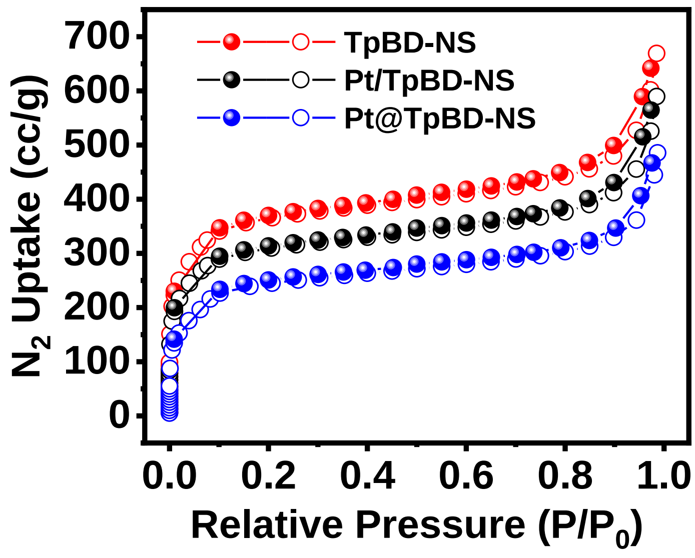

Supplementary Figure 9. Comparison of Nitrogen sorption curves of TpBD-NS based materials measured at 77 K.

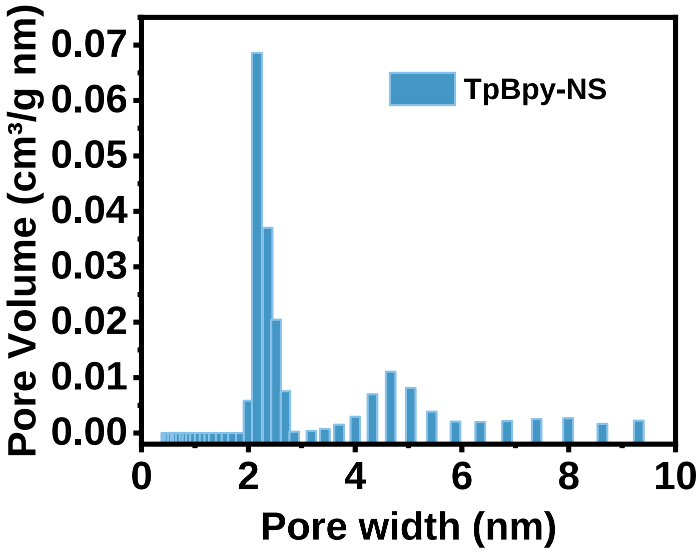

Supplementary Figure 10. The pore-size distribution profiles for the TpBpy-NS.

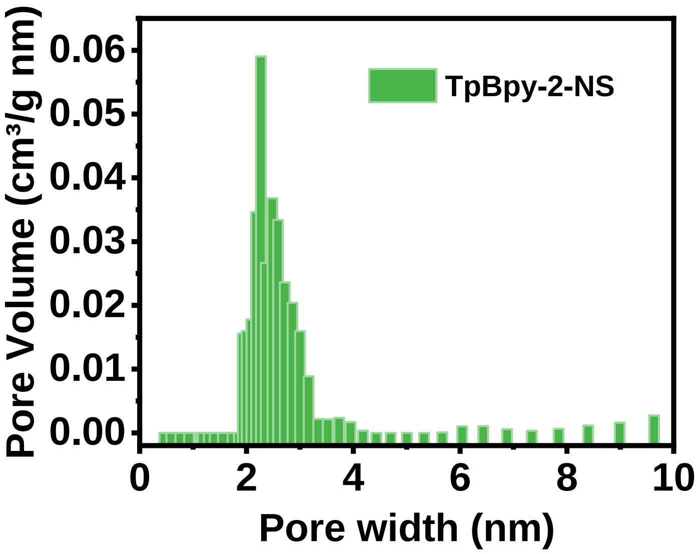

Supplementary Figure 11. The pore-size distribution profiles for the TpBpy-2-NS.

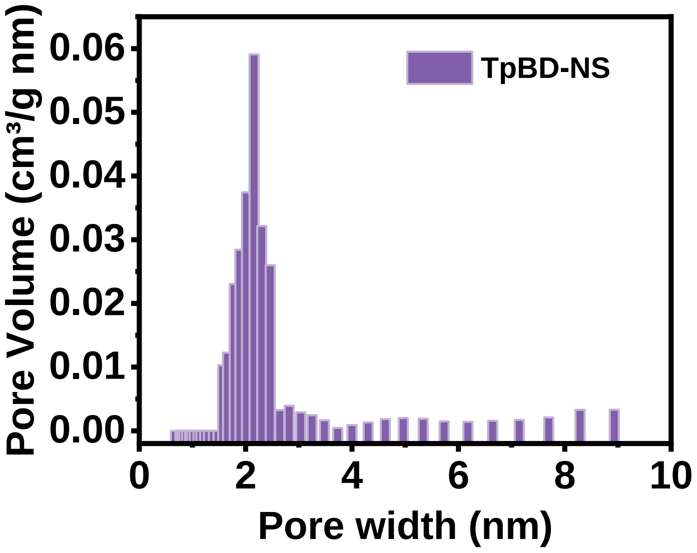

Supplementary Figure 12. The pore-size distribution profiles for the TpBD-NS.

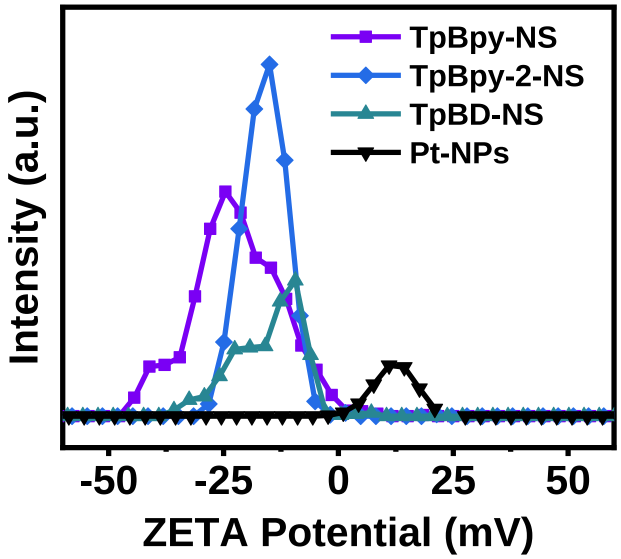

Supplementary Figure 13. ZETA potential of TpBpy-NS, TpBpy-2-NS, TpBD-NS and Pt NPs.

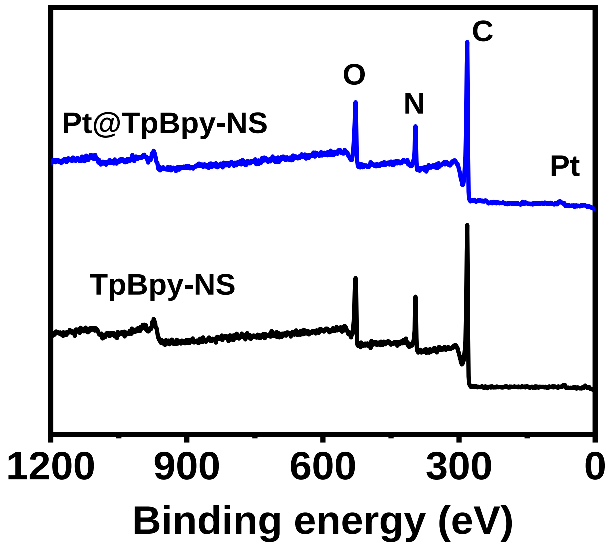

Supplementary Figure 14. Survey scan XPS profiles of TpBpy-NS and Pt@TpBpy-NS.

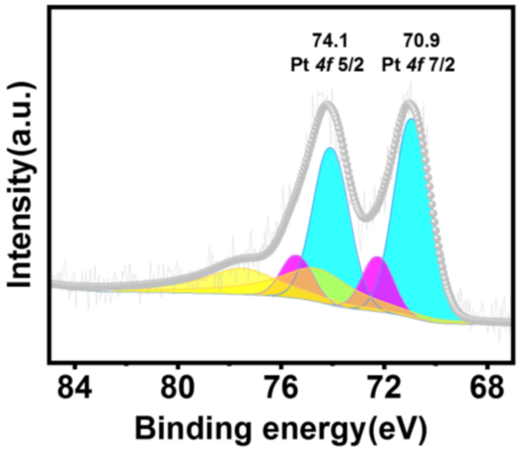

Supplementary Figure 15. Pt *4f* XPS spectra of Pt@TpBpy-NS.

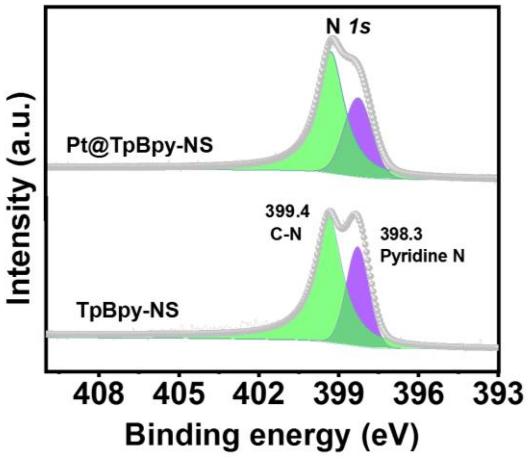

Supplementary Figure 16. N *1s* XPS spectra of TpBpy-NS and Pt@TpBpy-NS.

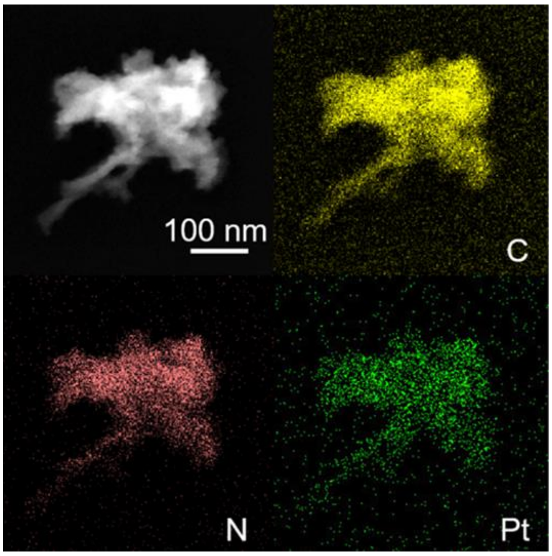

Supplementary Figure 17. TEM EDX mapping of Pt@TpBpy-NS.

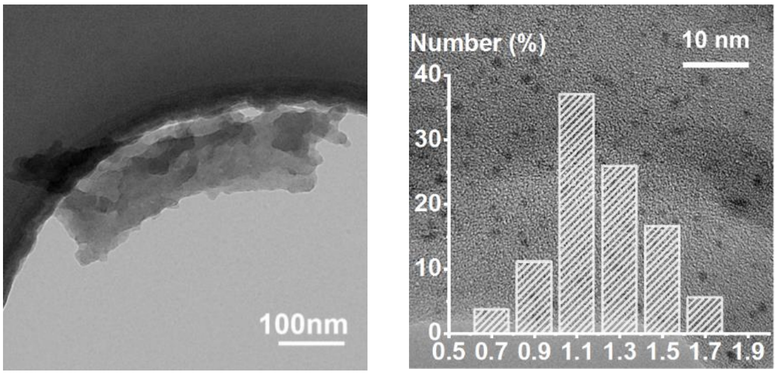

Supplementary Figure 18. TEM image of Pt@TpBpy-NS and the statistics of particle size distribution.

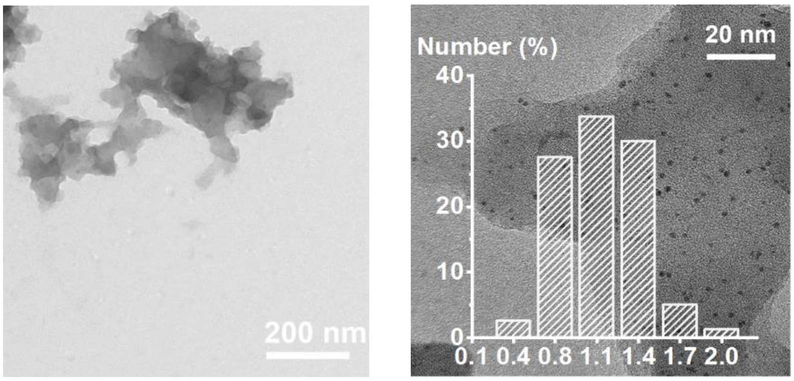

Supplementary Figure19. TEM image of Pt@TpBpy-2-NS and the statistics of particle size distribution.

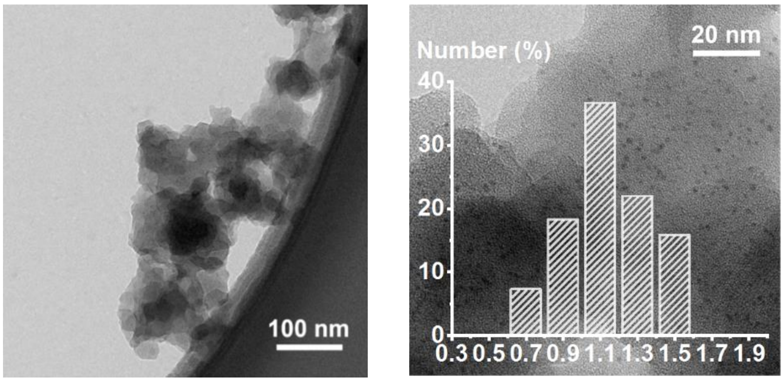

Supplementary Figure 20. TEM image of Pt@TpBD-NS and the statistics of particle size distribution.

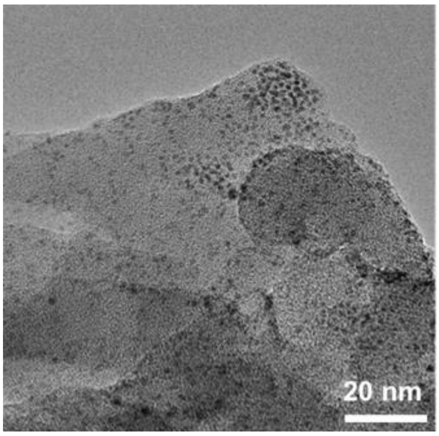

Supplementary Figure 21. TEM image of Pt/TpBpy-NS.

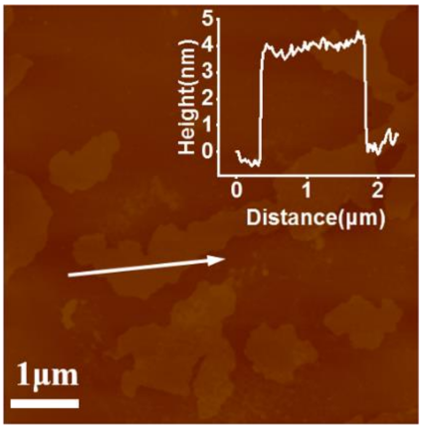

Supplementary Figure 22. AFM image of Pt@TpBpy-2-NS.

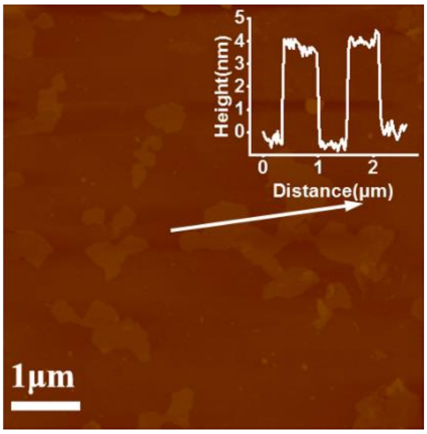

Supplementary Figure 23. AFM image of Pt@TpBD-NS.

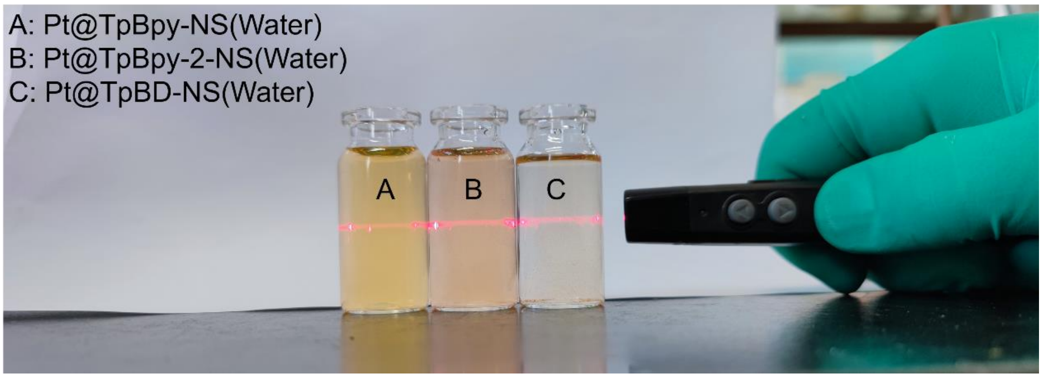

Supplementary Figure 24. Digital photo of the Tyndall effect.

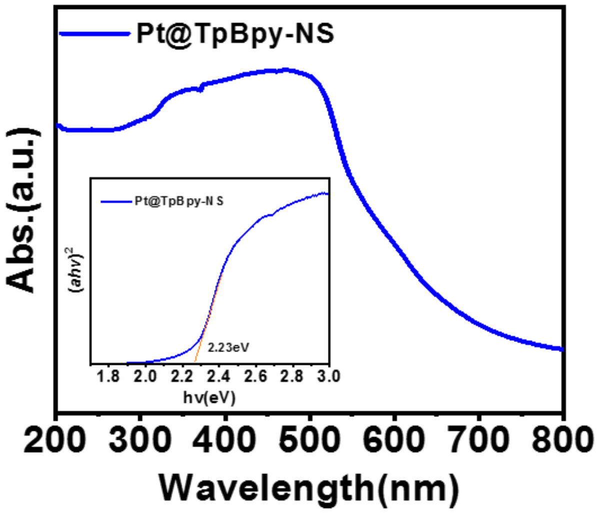

Supplementary Figure 25. The UV-vis DRS spectra of Pt@TpBpy-NS, inset Tauc plot for band gap calculation.

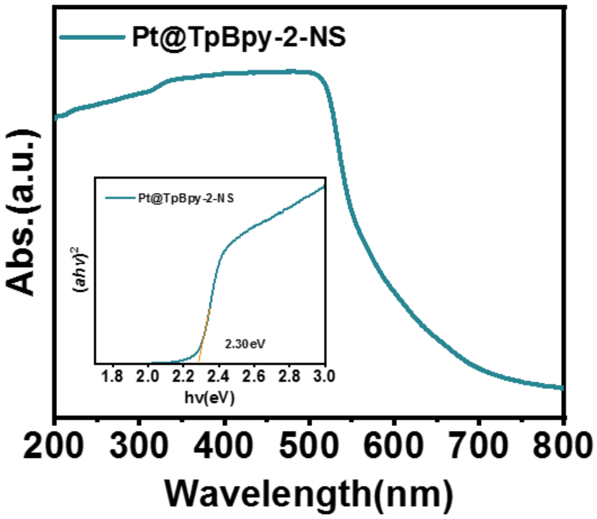

Supplementary Figure 26. The UV-vis DRS spectra of Pt@TpBpy-2-NS, inset Tauc plot for band gap calculation.

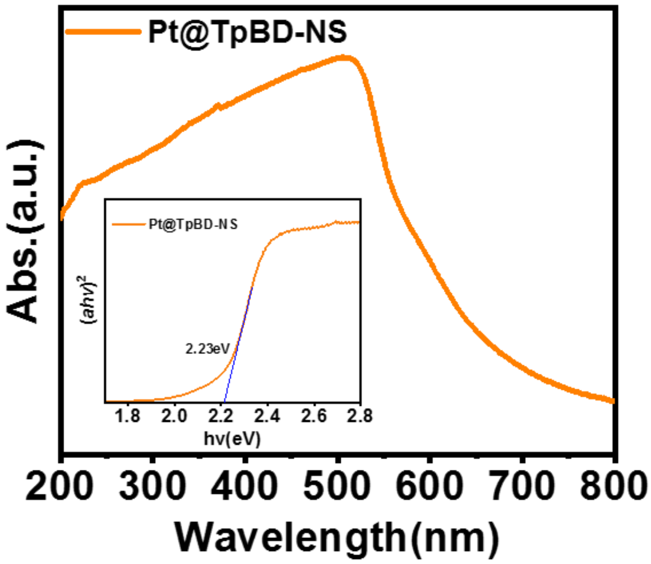

Supplementary Figure 27. The UV-vis DRS spectra of Pt@TpBD-NS, inset Tauc plot for band gap calculation.

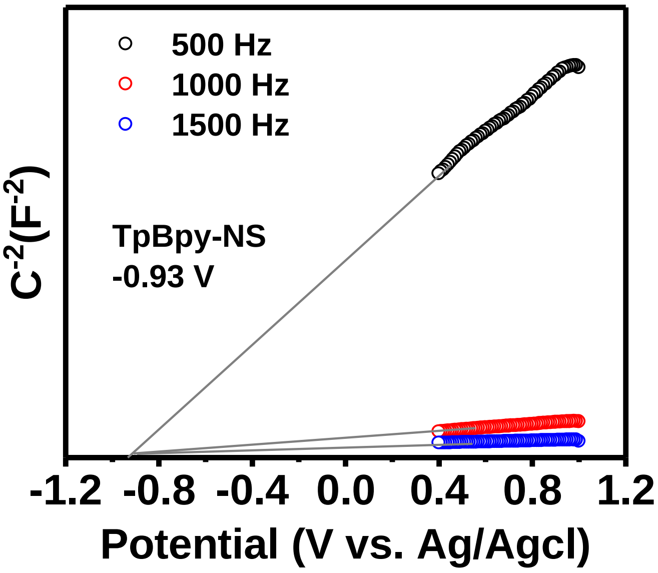

Supplementary Figure 28. Mott-Schottky plots of TpBpy-NS.

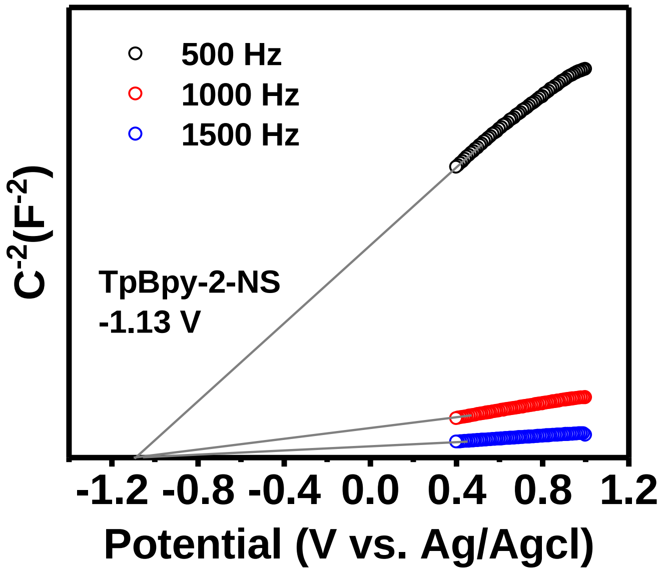

Supplementary Figure 29. Mott-Schottky plots of TpBpy-2-NS.

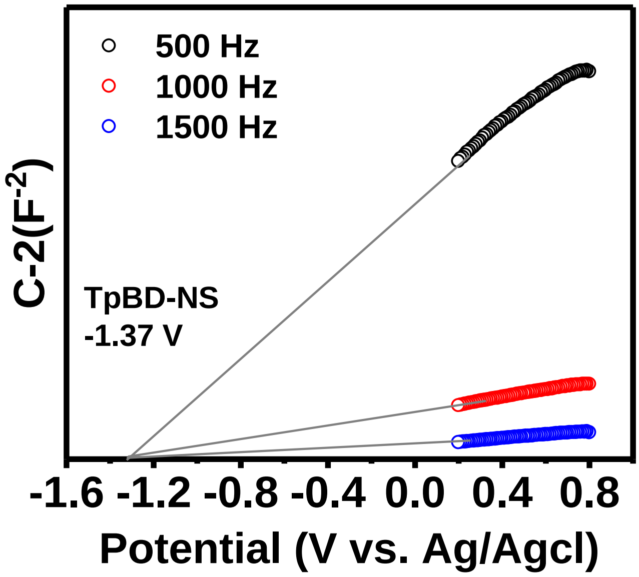

Supplementary Figure 30. Mott-Schottky plots of TpBD-NS.

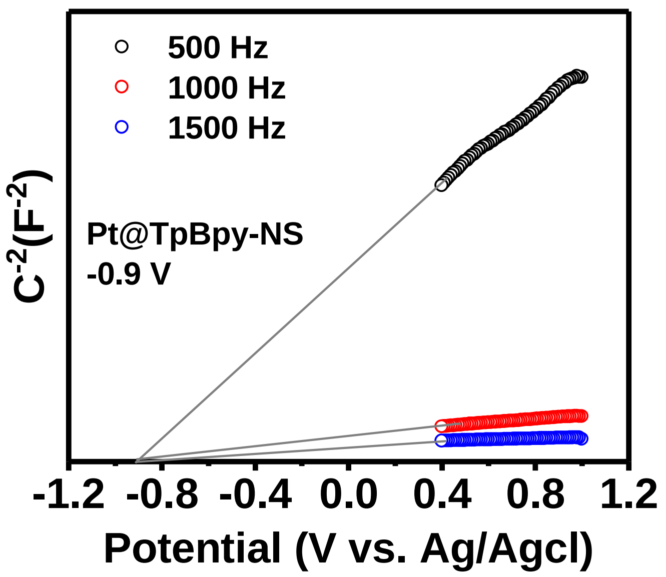

Supplementary Figure 31. Mott-Schottky plots of Pt@TpBpy-NS.

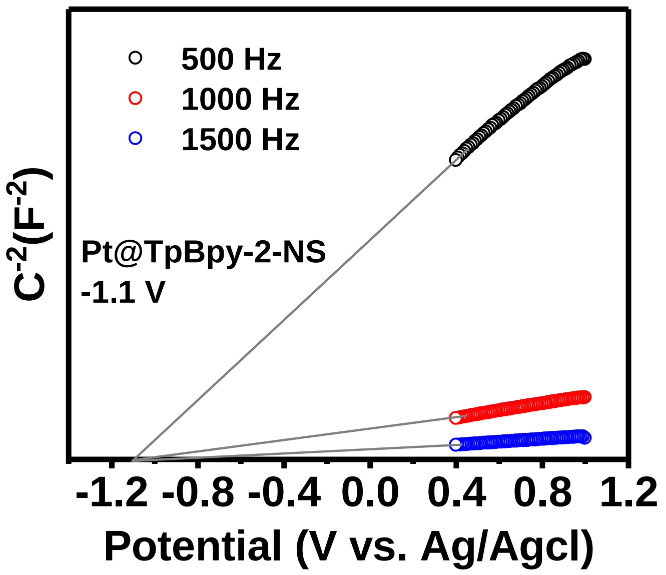

Supplementary Figure 32. Mott-Schottky plots of Pt@TpBpy-2-NS.

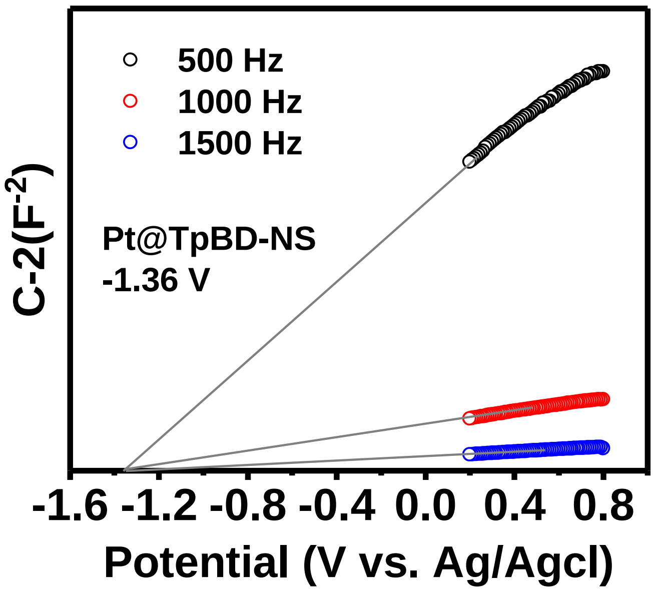

Supplementary Figure 33. Mott-Schottky plots of Pt@TpBD-NS.

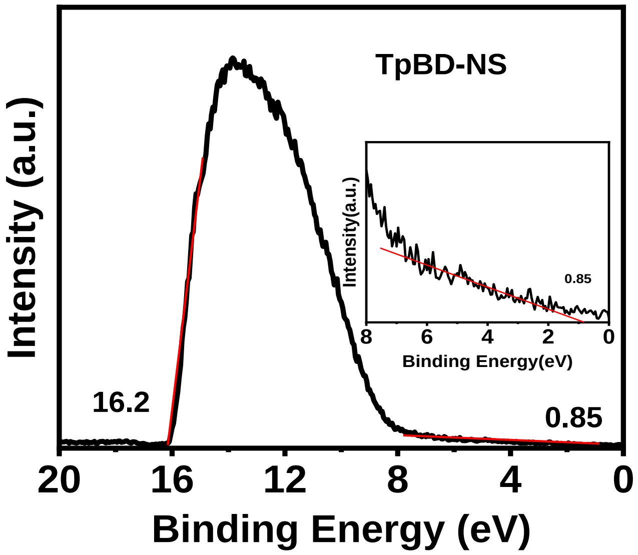

Supplementary Figure 34. The UPS spectra of TpBD-NS.

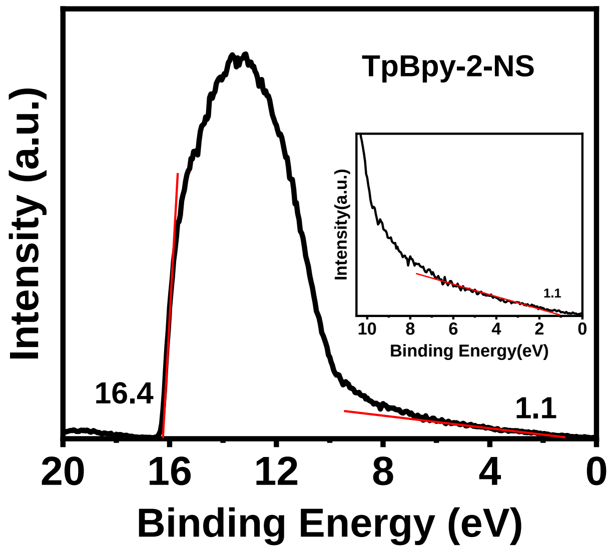

Supplementary Figure 35. The UPS spectra of TpBpy-2-NS.

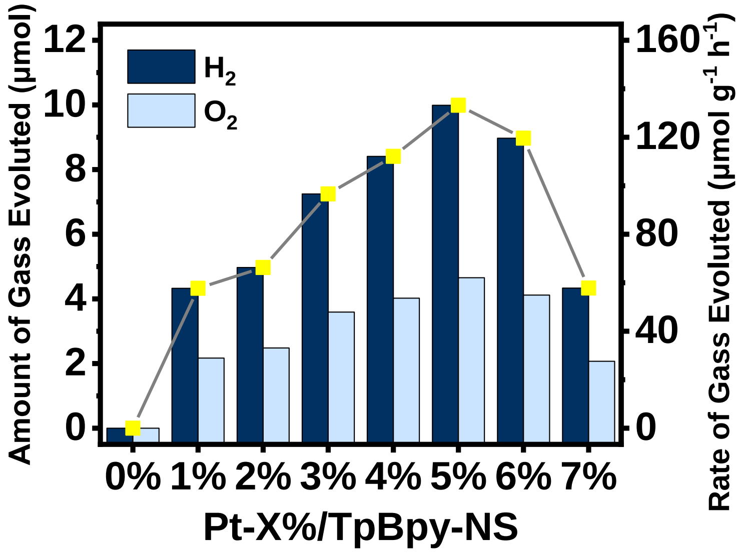

Supplementary Figure 36. The photocatalytic overall water splitting activities over Pt(*X*%)@TpBpy-NS (X = 1, 2, 3, 4, 5, 6, 7).

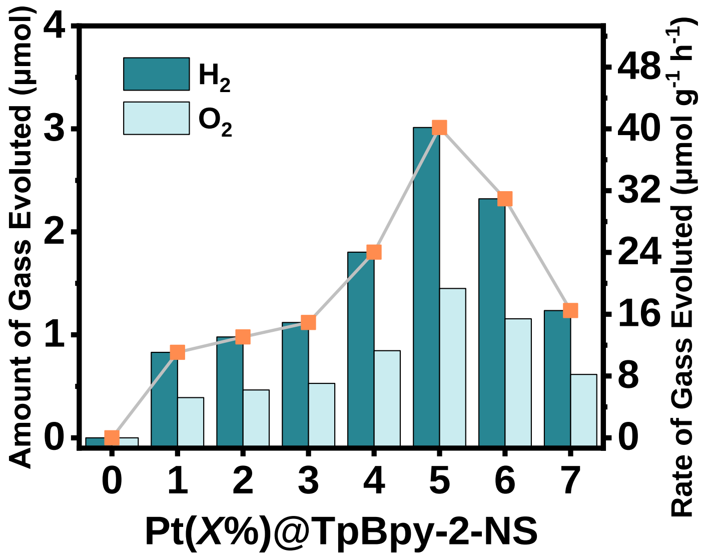

Supplementary Figure 37. The photocatalytic overall water splitting activities over Pt(*X*%)@TpBpy-2-NS (X = 1, 2, 3, 4, 5, 6, 7).

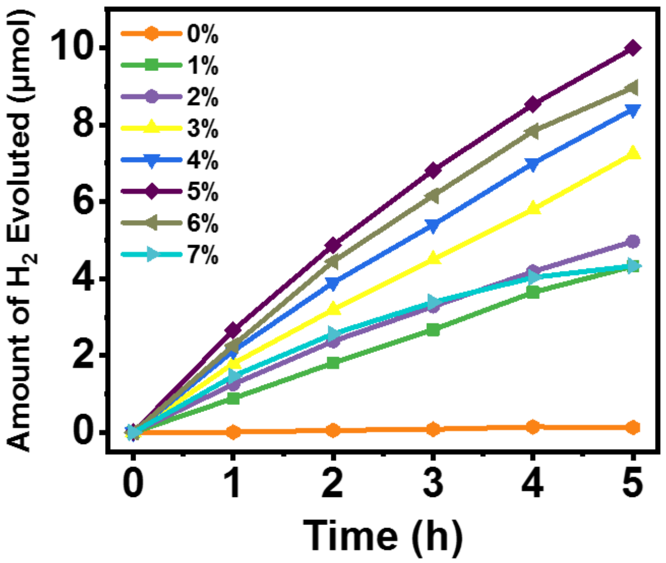

Supplementary Figure 38. The photocatalytic overall water splitting hydrogen evolution raters of Pt(*X*%)@TpBpy-NS as catalyst.

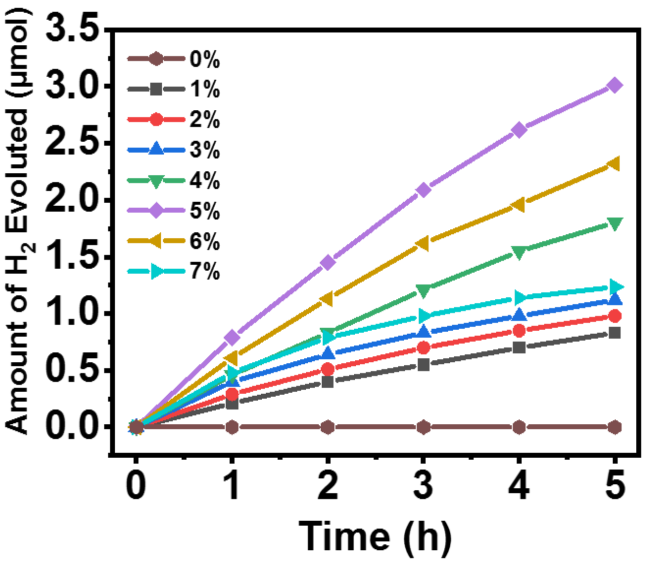

Supplementary Figure 39. The photocatalytic overall water splitting hydrogen evolution rates of Pt(*X*%)@TpBpy-2-NS as catalyst.

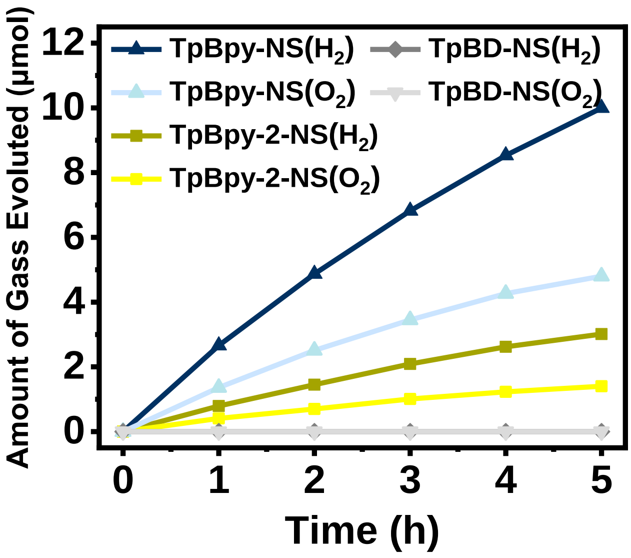

Supplementary Figure 40. The overall water splitting rate over Pt(*X*%)@COFs-NS.

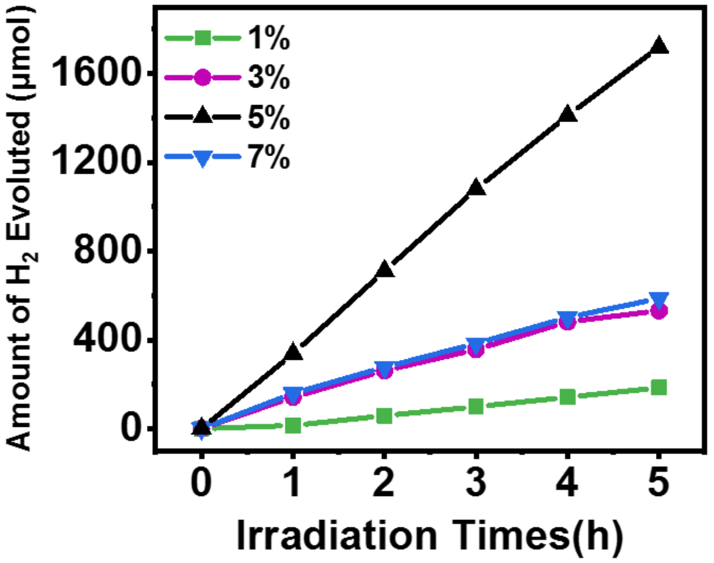

Supplementary Figure 41. The photocatalytic hydrogen evolution half-reaction rates of Pt(*X*%)@TpBpy-NS as catalyst.

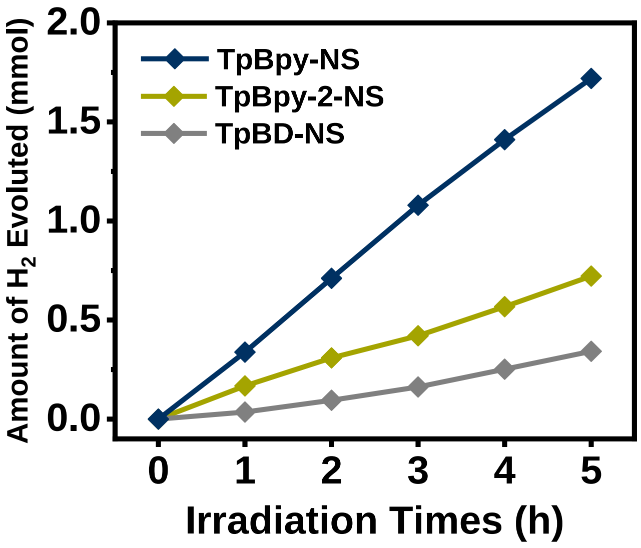

Supplementary Figure 42. The H_2 evolution half-reaction rate of Pt@COFs-NS.

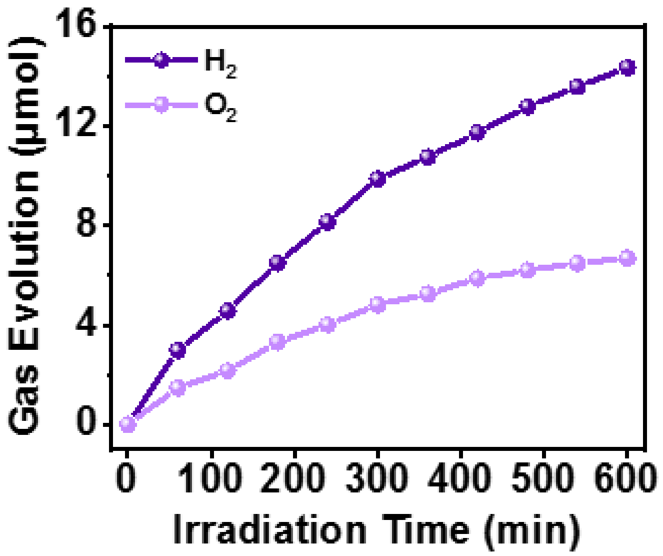

Supplementary Figure 43. The successive overall water splitting activity of Pt@TpBpy-NS within 600 min.

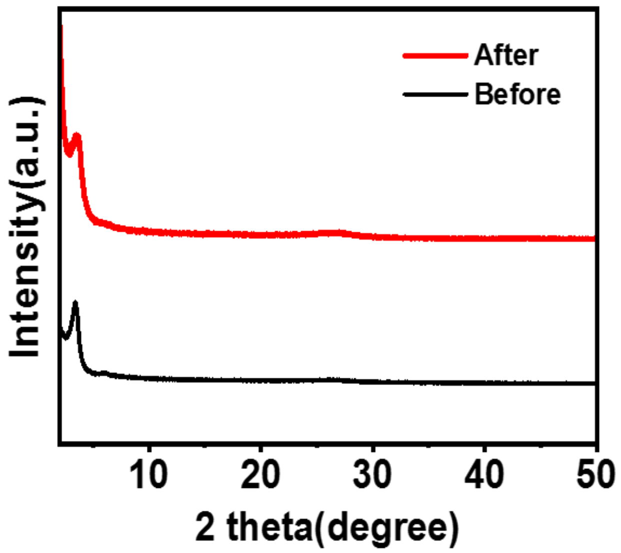

Supplementary Figure 44. Experimental XRD patterns of Pt@TpBpy-NS after overall water splitting reaction.

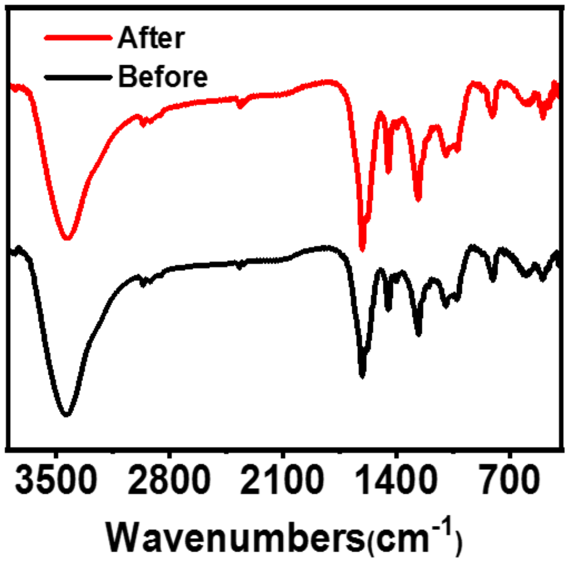

Supplementary Figure 45. The FT-IR spectra of Pt@TpBpy-NS after overall water splitting reaction.

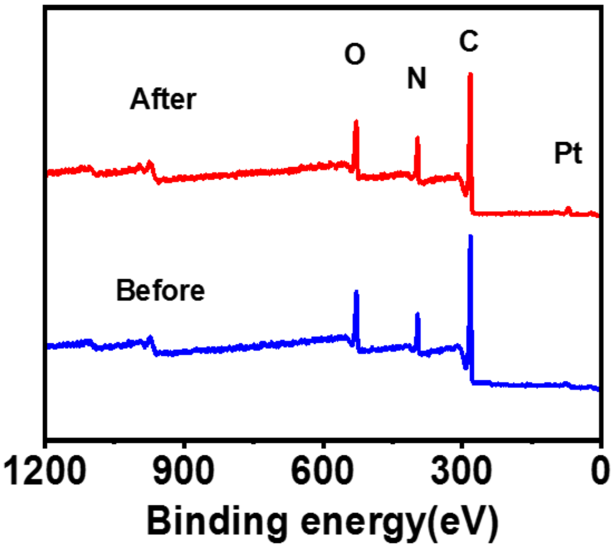

Supplementary Figure 46. Survey scan XPS profiles of Pt@TpBpy-NS after overall water splitting reaction.

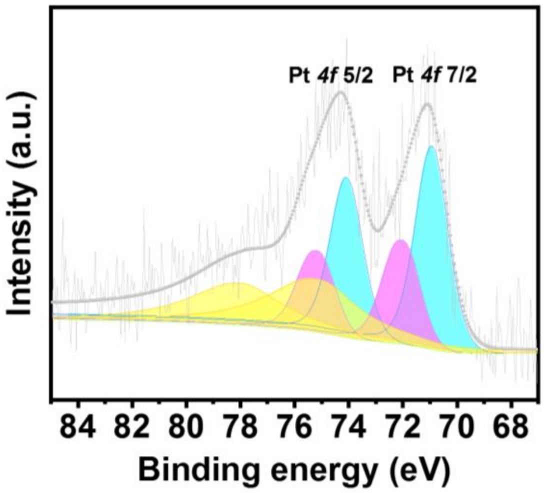

Supplementary Figure 47. High resolution Pt *4f* XPS profiles of Pt@TpBpy-NS after overall water splitting reaction.

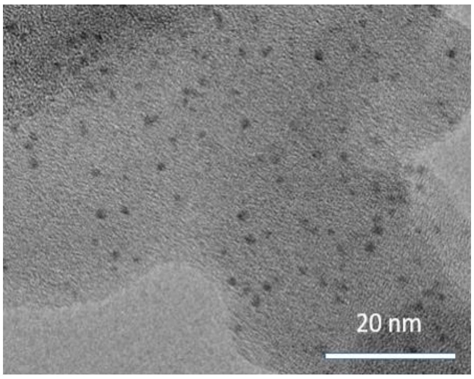

Supplementary Figure 48. TEM image of Pt@TpBD-NS after overall water splitting reaction.

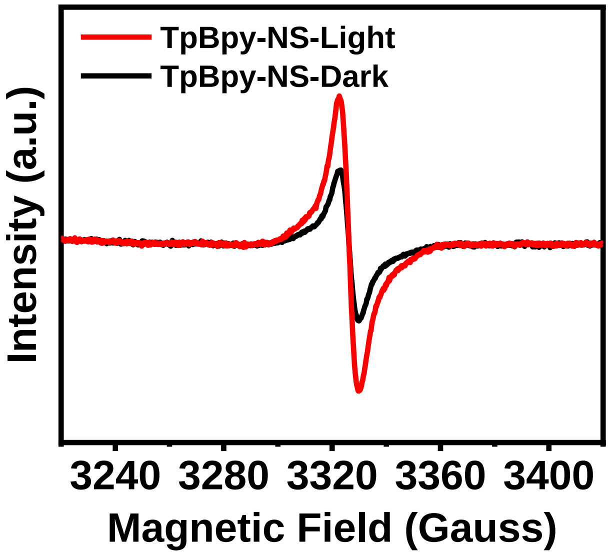

Supplementary Figure 49. EPR spectra of TpBpy-NS with or without light-irradiation.

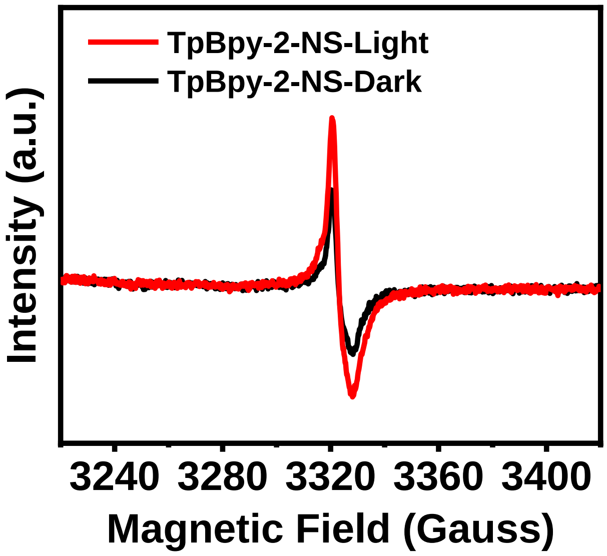

Supplementary Figure 50. EPR spectra of TpBpy-2-NS with or without the light-irradiation.

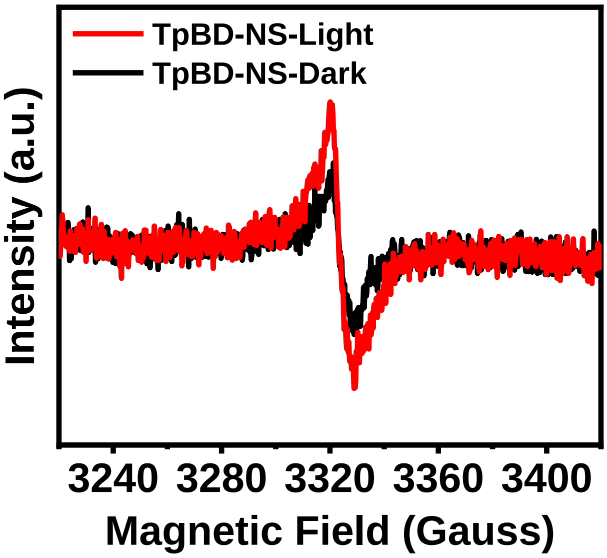

Supplementary Figure 51. EPR spectra of TpBD-NS with or without light-irradiation.

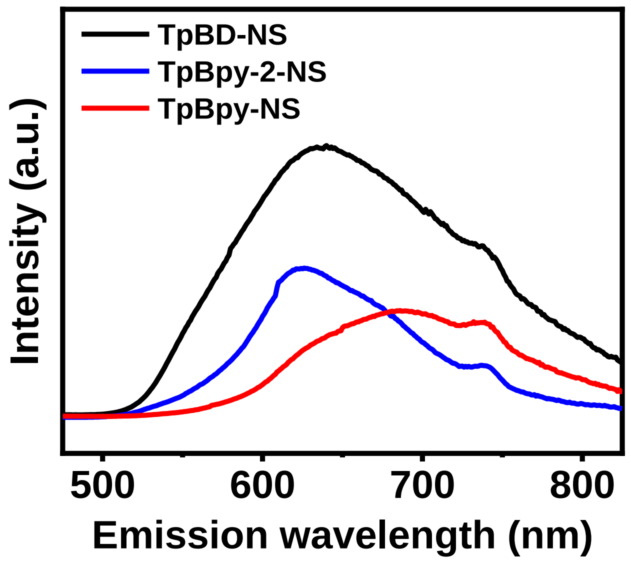

Supplementary Figure 52. The PL spectra of TpBD-NS, TpBpy-2-NS and TpBpy-NS.

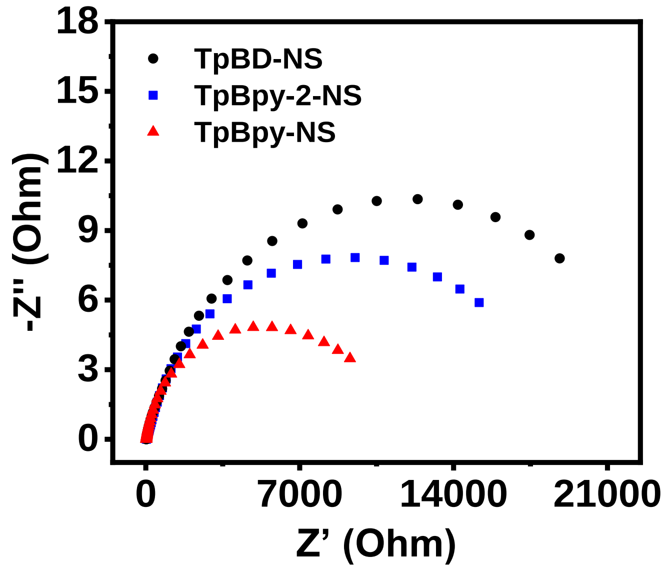

Supplementary Figure 53. The EIS spectra of TpBD-NS, TpBpy-2-NS and TpBpy-NS.

.

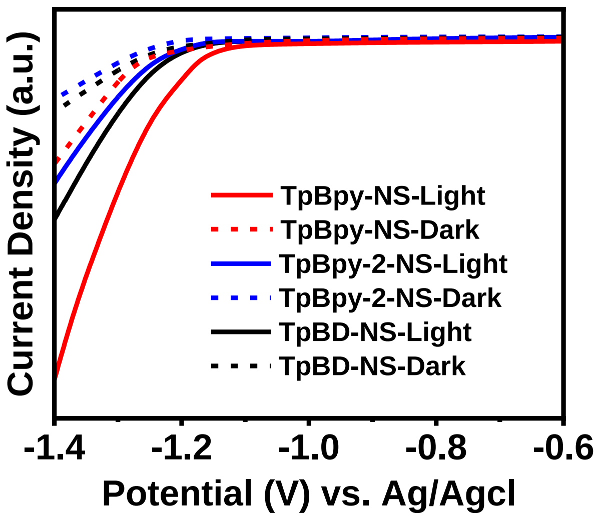

Supplementary Figure 54. The I–V curves in dark and under light irradiation of TpBD-NS, TpBpy-2-NS and TpBpy-NS (corresponds to the H_2 evolution).

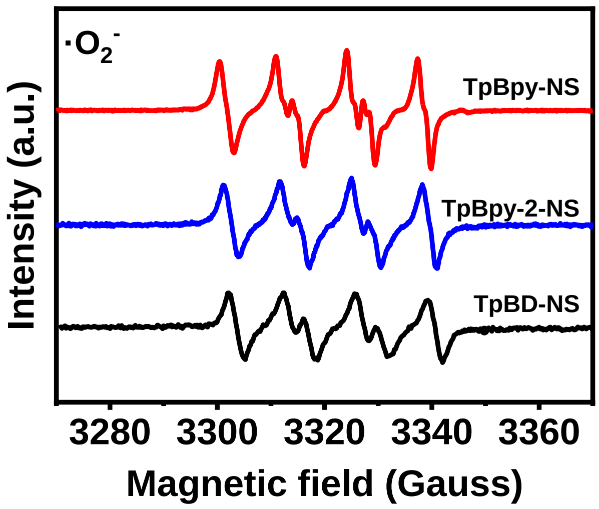

Supplementary Figure 55. EPR spectra of $\bullet O_2^-$ radical trapped by DMPO over TpBpy-NS, TpBpy-2-NS, and TpBD-NS samples for 3 min.

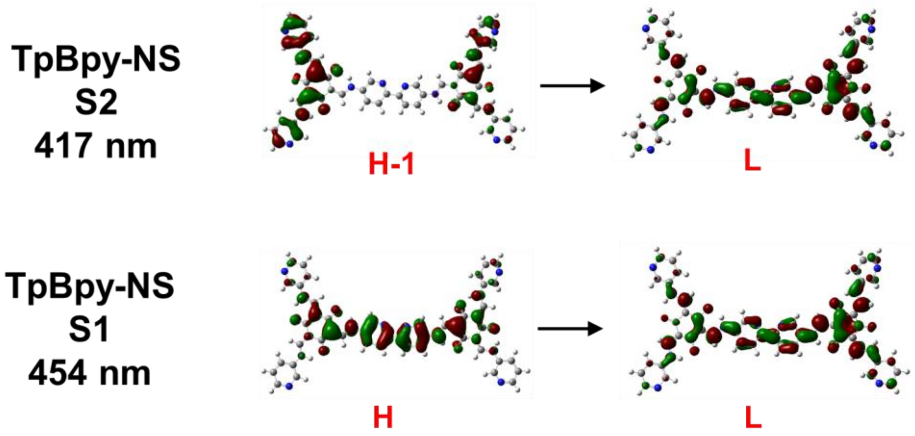

Figure S56. The TD-DFT calculated electronic transition of TpBpy-NS.

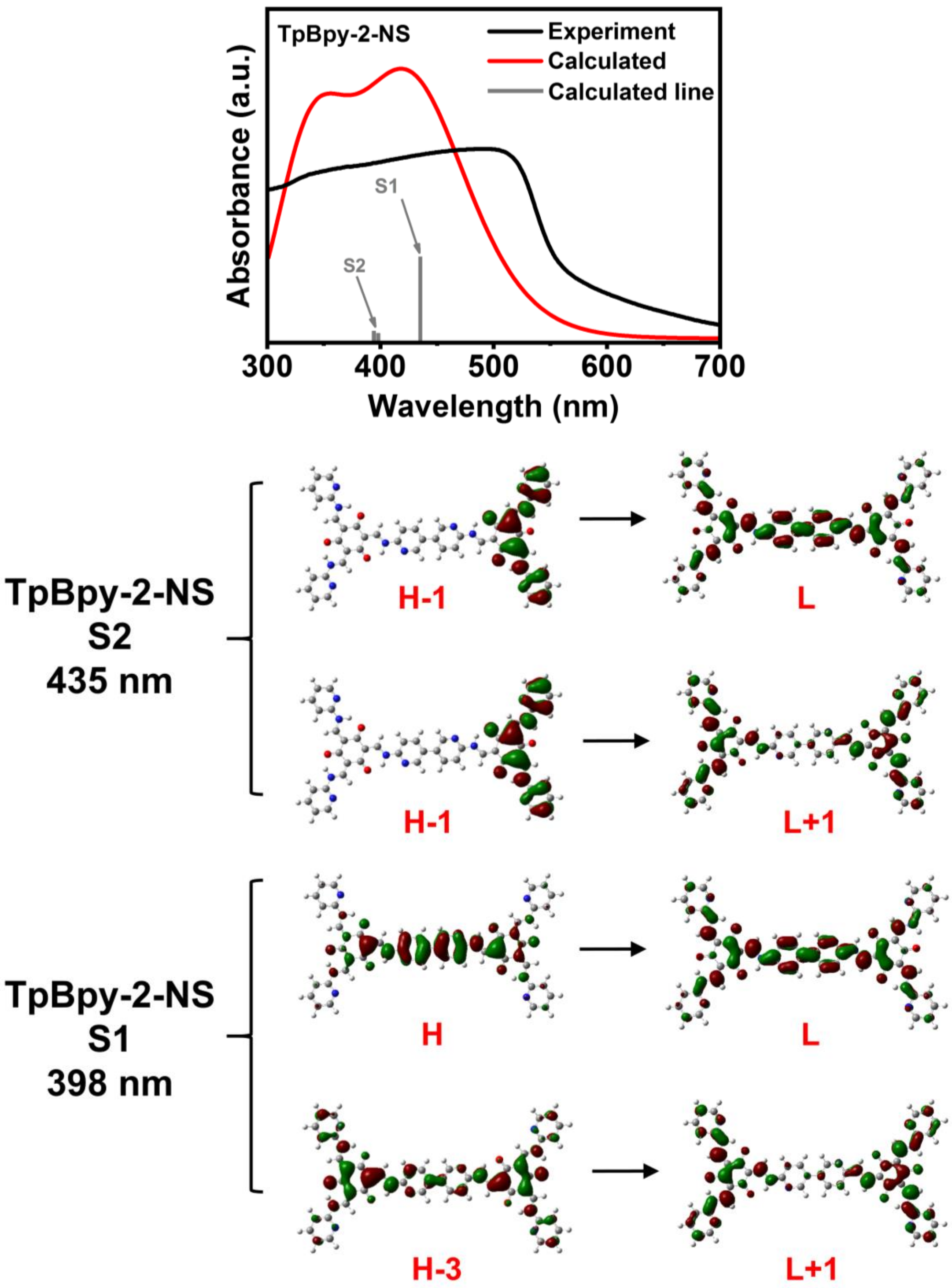

Supplementary Figure 57. The TD-DFT calculated electronic transition of TpBpy-2-NS.

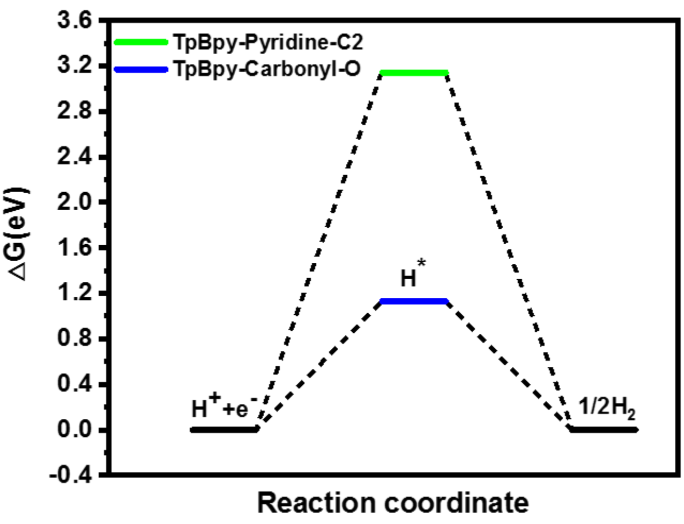

Supplementary Figure 58. The calculated Gibbs free energy change of intermediate states involved in HER processes for TpBpy-NS

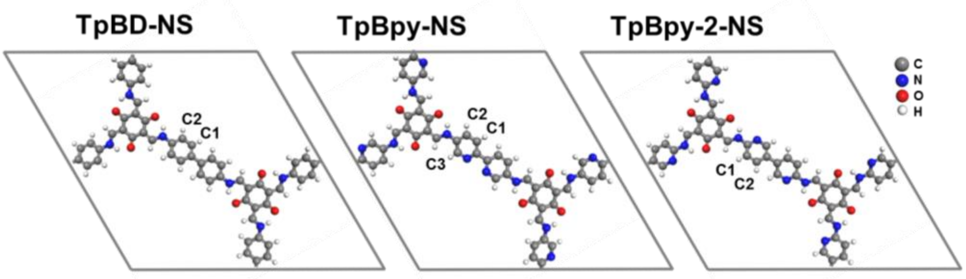

Supplementary Figure 59. The optimized structures of TpBD-NS, TpBpy-2-NS and TpBpy-NS.

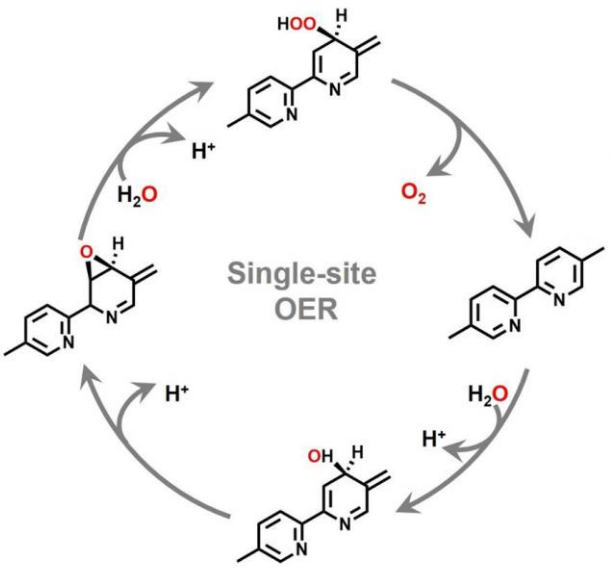

Supplementary Figure 60. The possible processe of OER reaction via single-site processe on bpy segment in TpBpy-NS.

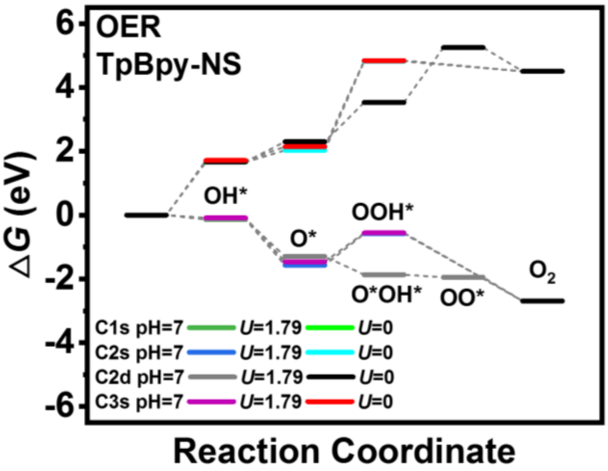

Supplementary Figure 61. The calculated Gibbs free energy change of intermediate states involved in OER processes for TpBpy-NS.

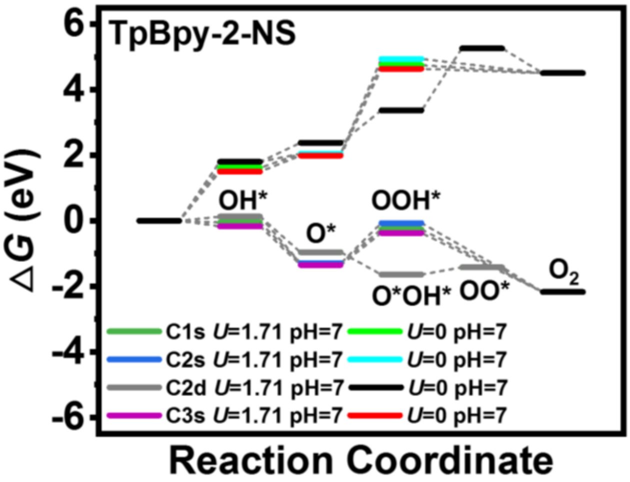

Supplementary Figure 62. The calculated Gibbs free energy change of intermediate states involved in OER processes for TpBpy-2-NS.

Supplementary Table 1. The Pt element contain analysis through ICP-OES. Content %= m(Pt)/m(Sample) ×100 %.

Sample	Element	Found.(wt %)
Pt(5%)@TpBpy-NS	Pt	1.23
Pt(5%)@TpBpy-2-NS	Pt	1.28
Pt(5%)@TpBD-NS	Pt	1.19
Pt(5%)@TpBpy-NS-after reaction	Pt	1.21

Supplementary Table 2. Comparison of overall water splitting performance with literature reports

Catalysts	Co-catalyst	Light Source	H_2 evolution rate (μmol g^{-1} h^{-1})	O_2 evolution rate (μmol g^{-1} h^{-1})	Ref.
Pt@TpBpy-NS	1.23 wt.% Pt.	300 W Xe Lamp $\lambda \geq 420$ nm	132	64	This Work
Pt@TpBpy-2-NS	1.28 wt.% Pt.	300 W Xe Lamp $\lambda \geq 420$ nm	41	19	This Work
CTF-0	3 wt.% Pt, 6 wt.%Co_3O_4	300 W Xe Lamp	82	40	1
Pt/g-C_3N_4	3wt.%Pt 1wt% CoO_x	300 W Xe lamp $\lambda \geq 420$ nm	6	3	2
Sea-urchin-structure g-C_3N_4	3wt.%Pt	300 W Xe lamp $\lambda \geq 420$ nm	41.5	20.3	3
C_3N_4/MnO_2	–	300 W Xe lamp $\lambda \geq 420$ nm	55.3	27.9	4
3D g-C_3N_4 NS	1wt.%Pt 3wt.% IrO_2	300 W Xe lamp $\lambda \geq 420$ nm	101.4	49.1	5
g-C_3N_4-Carbon Dots	–	300W Xe lamp $\lambda \geq 420$ nm	5	2.5	6
Co_1phosphide/PCN	–	300W Xe lamp $\lambda \geq 420$ nm	125	65	7
Ta_3N_5/BTON	0.3 wt% Pt 0.45wt% PtO_x/WO_3	300 W Xe Lamp $\lambda \geq 420$ nm	16	8	8
MnO_2/Monolayer g-C_3N_4	3wt.% Pt	300W Xe lamp $\lambda > 400$nm	60.6	28.9	9
CoO/g-C_3N_4	10wt.% CoO	LED $\lambda \geq 400$ nm	5.8	2.6	10
CoO/g-C_3N_4	30wt.% CoO	LED $\lambda \geq 400$ nm	50.2	27.8	11
Pt/CoP/g-C_3N_4	3wt.%Pt 3wt.% CoP	300 W Xe lamp $\lambda \geq 300$nm	26.3	12.5	12
g-C_3N_4 NWBs	1wt.% Pt	300 W Xe lamp, $\lambda \geq 300$nm	72	35.6	13
TiO_2/g-C_3N_4-WO_3	1% PtO_X	150W Xe lamp $\lambda > 200$ nm	29.4	14.3	14
C_3N_4-rGO-WO_3	1wt.% Pt	250W metal halide lamp, $\lambda \geq 420$nm	14.2	7.3	15

Supplementary References

1. Kong, D. et al. Stable complete water splitting by covalent triazine-based framework CTF-0. Chemcatchem 12, 2708-2712 (2020).
2. Zhang, G. et al. Overall water splitting by Pt/gC_3N_4 photocatalysts without using sacrificial agents. *Chem. Sci.* **7**, 3062–3066 (2016).
3. Zeng, Y. et al. Sea-urchin-structure g-C_3N_4 with narrow bandgap (~2.0 eV) for efficient overall water splitting under visible light irradiation. *Appl. Catal., B-Environ.* **249**, 275–281 (2019).
4. Liu, J. et al. A critical study of the generality of the two step two electron pathway for water splitting by application of a C_3N_4/MnO_2 photocatalyst. *Nanoscale* **8**, 11956–11961 (2016).
5. Chen, X. et al. Three-dimensional porous g-C_3N_4 for highly efficient photocatalytic overall water splitting. *Nano Energy* **59**, 644–650 (2019).
6. Qu, D. et al. Peering into water splitting mechanism of g-C_3N_4-carbon dots metal-free photocatalyst. *Appl. Catal., B-Environ.* **227**, 418–424 (2018).
7. Liu, W. et al. Single-site active cobalt-based photocatalyst with a long carrier lifetime for spontaneous overall water splitting. *Angew. Chem., Int. Ed.* **56**, 9312–9317 (2017).
8. Dong, B. et al. Heterostructure of 1D Ta_3N_5 nanorod/$BaTaO_2N$ nanoparticle fabricated by a one-step ammonia thermal route for remarkably promoted solar hydrogen production. *Adv. Mater.* **31**, 1808185 (2019).
9. Mo, Z. et al. Construction of MnO_2/Monolayer g-C_3N_4 with Mn vacancies for Z-scheme overall water splitting. *Appl. Catal., B-Environ.* **241**, 452–460 (2019).
10. Han, M. et al. One-step synthesis of CoO/g-C_3N_4 composites by thermal decomposition for overall water splitting without sacrificial reagents. *Inorg. Chem. Front.* **4**, 1691–1696 (2017).
11. Guo, F. et al. CoO and g-C_3N_4 complement each other for highly efficient overall water splitting under visible light. *Appl. Catal., B-Environ.* **226**, 412–420 (2018).
12. Pan, Z. et al. Decorating CoP and Pt nanoparticles on graphitic carbon nitride nanosheets to promote overall water splitting by conjugated polymers. *ChemSusChem* **10**, 87–90 (2017).
13. Zhang, K. et al. Tunable bandgap energy and promotion of H_2O_2 oxidation for overall water splitting from carbon nitride nanowire bundles. *Adv. Energy Mater.* **6**, 1502352 (2016).
14. Yan, J. et al. Fabrication of TiO_2/C_3N_4 heterostructure for enhanced photocatalytic Z-scheme overall water splitting. *Appl. Catal., B-Environ.* **191**, 130–137 (2016).
15. Zhao, G. et al. Facile structure design based on C_3N_4 for mediator-free Z-scheme water splitting under visible light. *Catal.: Sci. Technol.* **5**, 3416–3422 (2015).
