# Supplementary material for: Engineering β-ketoamine covalent organic frameworks for photocatalytic overall water splitting
Source: Nat Commun. 2023 Feb 3;14:593. doi: 10.1038/s41467-023-36338-x (PMC9898260; doi:10.1038/s41467-023-36338-x)
Supplement: Supplementary file 2 — Description of Additional Supplementary Files [file 41467_2023_36338_MOESM2_ESM.docx]

**Legends for Supplementary Data 1**
**Description**：Atomic coordinates of the computational TpBpy-NS, TpBpy-2-NS and TpBD-NS structures; standard orientation of TpBpy-NS and TpBpy-2-NS model structure calculated by Gaussian.
